# Crosstalks between Myo-Inositol Metabolism, Programmed Cell Death and Basal Immunity in *Arabidopsis*

**DOI:** 10.1371/journal.pone.0007364

**Published:** 2009-10-08

**Authors:** Ping Hong Meng, Cécile Raynaud, Guillaume Tcherkez, Sophie Blanchet, Kamal Massoud, Séverine Domenichini, Yves Henry, Ludivine Soubigou-Taconnat, Caroline Lelarge-Trouverie, Patrick Saindrenan, Jean Pierre Renou, Catherine Bergounioux

**Affiliations:** 1 Institut de Biotechnologie des Plantes, UMR CNRS 8618, Université Paris-Sud XI, bât 630, Plateau de Moulon, Orsay, France; 2 Plateforme Métabolisme Métabolome IFR87, Institut de Biotechnologie des Plantes, Université Paris-Sud XI, bât 630, Plateau du Moulon, Orsay, France; 3 Unité de Recherche en Génomique Végétale, 2, CP5708, Evry, France; Ecole Normale Superieure, France

## Abstract

**Background:**

Although it is a crucial cellular process required for both normal development and to face stress conditions, the control of programmed cell death in plants is not fully understood. We previously reported the isolation of ATXR5 and ATXR6, two PCNA-binding proteins that could be involved in the regulation of cell cycle or cell death. A yeast two-hybrid screen using ATXR5 as bait captured AtIPS1, an enzyme which catalyses the committed step of myo-inositol (MI) biosynthesis. *atips1* mutants form spontaneous lesions on leaves, raising the possibility that MI metabolism may play a role in the control of PCD in plants. In this work, we have characterised *atips1* mutants to gain insight regarding the role of MI in PCD regulation.

**Methodology/Principal Findings:**

- lesion formation in *atips1* mutants depends of light intensity, is due to PCD as evidenced by TUNEL labelling of nuclei, and is regulated by phytohormones such as salicylic acid - MI and galactinol are the only metabolites whose accumulation is significantly reduced in the mutant, and supplementation of the mutant with these compounds is sufficient to prevent PCD - the transcriptome profile of the mutant is extremely similar to that of lesion mimic mutants such as *cpr5*, or wild-type plants infected with pathogens.

**Conclusion/Significance:**

Taken together, our results provide strong evidence for the role of MI or MI derivatives in the regulation of PCD. Interestingly, there are three isoforms of IPS in *Arabidopsis*, but AtIPS1 is the only one harbouring a nuclear localisation sequence, suggesting that nuclear pools of MI may play a specific role in PCD regulation and opening new research prospects regarding the role of MI in the prevention of tumorigenesis. Nevertheless, the significance of the interaction between AtIPS1 and ATXR5 remains to be established.

## Introduction

The decision whether a cell should live or die is fundamental to the survival of all organisms. In plants, Programmed Cell Death (PCD) is required both for normal development and to face stress conditions (for a review see [1]). One well characterised example of plant PCD is the hypersensitive response (HR), a localised cell death induced by pathogen attacks which allows confinement of the infection [2]. Many studies focusing either on the signalling pathways controlling PCD or on the cellular effectors, have improved our understanding of this process [1], [3]. Reactive oxygen species (ROS) such as H_2_O_2_ or O_2_^−^, as well as phytohormones such as salicylic acid (SA), jasmonic acid (JA) or ethylene appear to be key players for HR regulation [4]. Once PCD promoting signals are perceived by plant cells, effectors of the suicide programme are activated. In animal cells, the molecular bases of PCD are well described, but to date, plant homologues of mammalian core apoptosis regulators have been scarce [1]. Nevertheless, several mutants have been isolated that are affected in the control of PCD. Notably, about 40 lesion mimic mutants (LMM) have been described: these mutants form spontaneous lesions in the absence of pathogen challenge. Mutated genes in LMM could thus correspond to repressors of PCD (reviewed in [5]).

In the absence of clear sequence conservation between animals and plants, an alternative strategy to isolate PCD regulators or effectors is to search for functional homologues. This approach led to the identification of Caspase-like activities in plants (reviewed in [3]). In animal cells, the Proliferating Cells Nuclear Antigen (PCNA) plays a pivotal role in the regulation of cell proliferation: it functions as a processivity factor for DNA polymerase, but is also the target of regulatory proteins, including pro-apoptotic factors (reviewed in [6]). We previously isolated ATXR5 and ATXR6, two *Arabidopsis* SET (Suvar(3–9), Enhancer of zeste, Trithorax)-domain proteins for their ability to bind *Arabidopsis* PCNA [7]. These proteins are involved in histone methylation and heterochromatin formation [8]. Furthermore, we showed that their over-expression induced cell death respectively in pollen and anther endothetium [7], raising the possibility that they may function as positive regulators of PCD. We identified *Arabidopsis* myo-inositol synthase AtIPS1 as an interactor of ATXR5 (C. Raynaud, unpublished data).

Although it was first isolated from muscles, myo-inositol (MI) is a ubiquitous compound found in all living organisms. MI is synthesised from D-glucose in three steps: first glucose is phosphorylated by the hexokinase, second, glucose-6-P is converted to 1L-myo-Inositol-1-P by the 1L-myo-Inositol-1-Phosphate synthase (hereafter referred to as IPS), and finally, 1L-myo-Inositol-1-P is dephosphorylated by a phosphatase to produce free MI. The second step is the rate limiting step for MI biosynthesis in most organisms, including plants [9], [10]. IPS are well-conserved enzymes found both in eukaryotes and in prokaryotes. In plant cells, inositol derivatives play critical and diverse biological roles. These include phosphate storage in the form of phytic acid (an hexaphosphorylated form of MI), cell wall biogenesis, control of auxin physiology, membrane biogenesis, signal transduction and stress tolerance (for review see [10]). In salt-tolerant or cold-tolerant plant species, myo-inositol biosynthesis seems to play a pivotal role in protection mechanisms (reviewed in [11]). For example in ice plant (*Mesembryanthemum crystallinum*), an IPS homologue has clearly been demonstrated to participate in salt-stress tolerance [12]. Likewise, in *Spirodella polyrrhiza*, the expression of the *TUR1* gene, encoding IPS is induced in dormant buds (turions) in response to ABA and could be involved in their high tolerance to environmental stress such as salt stress [13]. However, such a role seems to highly depend on the species: over-expression of *TUR1* in *Arabidopsis* results in elevated levels of MI, but does not increase salt-stress tolerance [14].

Loss-of-function approaches have been conducted in several plant species to inactivate IPS. Most of these studies were aimed at reducing phytate contents in grains of crop plants, because phytate is detrimental for both human nutrition and the environment [15]. However, down-regulation of IPS activity is generally unfavourable for plant development: in maize (*Zea mays*), low phytic acid mutants display a reduction of seed dry weight [16] and in soybean (*Glycine max*), reduction of IPS expression with an RNAi strategy results in seed abortion [17]. Similarly, down-regulation of IPS *Solanum tuberosum* results in pleiotropic defects including reduced apical dominance, altered leaf morphology, decreased tuber yield and precocious senescence [18]. In *Arabidopsis*, *AtIPS1* was initially isolated based on its ability to complement a yeast mutant defective for the *INO1* gene [19]; AtIPS1 belongs to a family of three *IPS* genes. Recently, Murphy *et al.* reported that *Arabidopsis atips1* and *atips2* mutants display reduced phytic acid accumulation and that *atips2*, but not *atips1* is compromised in resistance to various pathogens [20].

Here, we identified AtIPS1 as an interacting partner of ATXR5 and ATXR6, two proteins that could play a role in the control of cell proliferation or cell death. In agreement with this hypothesis, *atips1* mutants displayed spontaneous lesions on rosette leaves. To gain insight on the control of cell death and more specifically on the role of MI biosynthesis in plants, we functionally characterised *AtIPS1*. We show that lesions formed on *atips1* leaves correspond to PCD and that *atips1* is likely a novel lesion mimic mutant (LMM). We discuss the plausible relationships between inositol metabolism and the control of PCD.

## Results

### AtIPS1 interacts with ATXR5 and ATXR6, and accumulates both in the cytoplasm and in the nucleus

To gain further insight into ATXR5 function, and to identify protein partners potentially involved in the control of PCD, a yeast two-hybrid screening was performed, leading to the identification of AtIPS1 (1-L-myo-inositol-1P synthase; E.C.5.5.1.4), a protein that could also bind ATXR6 (C. Raynaud, unpublished data). Mutant lines for *AtIPS1* were obtained. Plants mutated for *AtIPS1* exhibited spontaneous cell death when transferred to long day conditions to induce flowering. The isolated clone encompassed a truncated form of the AtIPS1 protein lacking the first 146 amino-acids. To confirm the interaction, yeast was re-transformed using this construct or a construct corresponding to the full length protein (summarized in Table 1). The short version of AtIPS1 was found to interact with ATXR6 and with a splicing variant of ATXR5 lacking its PCNA binding site [7]. The full length protein was capable to interact with itself or with the truncated form, consistent with the fact that the protein functions as trimers or tetramers [10]. By contrast, the full length protein could not interact with ATXR5 and ATXR6, suggesting that the N-terminus of the protein is not required for this interaction. It has been shown previously that truncated cDNAs detected valid two-hybrid interactions that were not seen when using full length ORFs [21]. The lack of interaction between ATXR5/6 and the full-length version of AtIPS1 might be due to the involvement of AtIPS1 in competing interactions with itself or even possibly with the yeast INO protein.

**Table 1:** AtIPS1 interacts with ATXR5 and ATXR6 in the yeast two-hybrid system.

	pGAD424	shAtIPS1-AD	flAtIPS1-AD	PCNA1-AD
pGBT-9	−	−	−	−
ATXR5-BD	−	+	−	+
ATXR6-BD	−	+	−	+
ATXR5▒ex2-BD	−	+	−	−
flAtIPS1-BD	−	+	+	nd

pGAD424 contains the activation domain of GAL4 (AD) while pGBT-9 contains its DNA-binding domain (BD). shAtIPS1: truncated form of AtIPS1 lacking its first 146 amino-acids, flAtIPS1: full length AtIPS1 protein. ATXR5Δex2 is encoded by a splicing variant of ATXR5 and lacks its PCNA binding domain [7].

**Figure 1:**
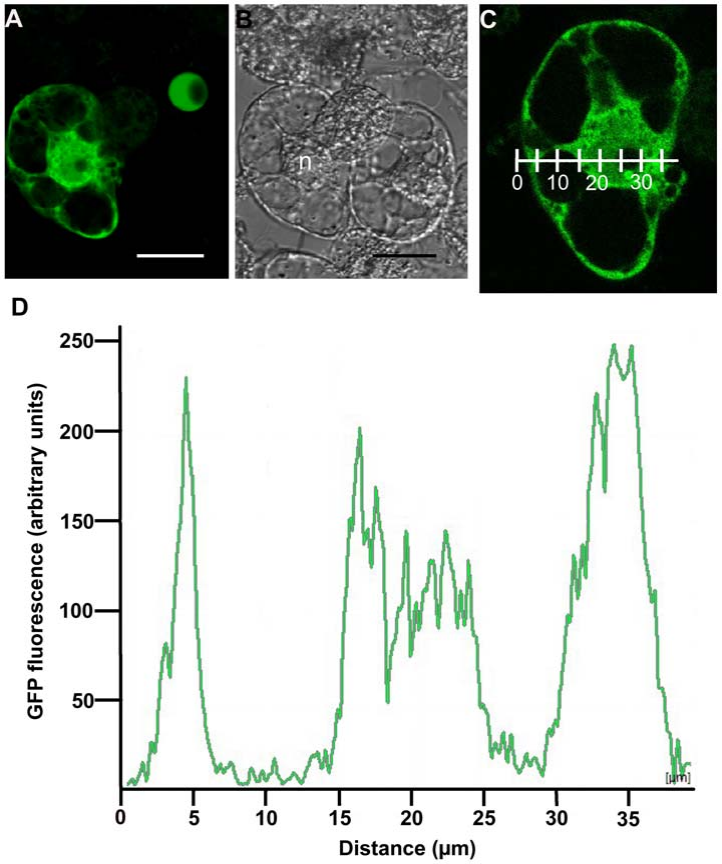
AtIPS1 accumulates in the nucleus and the cytoplasm of transiently transformed BY-2 cells. BY-2 protoplasts were transformed with a *PromAtIPS1::AtIPS1-GFP* construct. GFP fluorescence was observed 24 h later using a confocal microscope. (A) GFP fluorescence. (B) Transmission image. (C) Optical section used for GFP quantification. (D) GFP quantification on section shown on panel C. GFP was quantified along the graduated line. Scale bar  = 25 µm for panels A–B. On panel B, the position of the nucleus is indicated by the letter n.

Yeast two-hybrid forces targeting of proteins to the nucleus and can therefore lead to the identification of interactions between proteins that do not accumulate in the same cellular compartment. We therefore generated a construct encompassing a fusion between AtIPS1 and the Green Fluorescent Protein (GFP) to determine the sub-cellular localisation of AtIPS1. As shown on Figure 1 (A–C), AtIPS1 accumulated both in the cytoplasm and in the nucleus in transiently transformed BY-2 cells. To confirm that AtIPS1-GFP was found inside the nucleus and not at its periphery, GFP fluorescence was quantified in an optical section spanning the nucleus and the cytoplasm (Figure 1C–D). GFP fluorescence was indeed found in the nucleus, but excluded from the nucleolus, as is often the case for nuclear protein. This localisation is compatible with an interaction with ATXR5 and ATXR6, since these two proteins accumulate in the nucleus [7].

### Isolation of *atips1* mutants

Using the T-DNA express software (http://signal.salk.edu/cgi-bin/tdnaexpress), we identified two independent lines harboring an insertion in *AtIPS1*. In the *atips1-1* allele (SALK_023626; Col-0 background), the T-DNA was inserted at the end of the 4th intron. This line was also described by Murphy *et al* [20]. In the *atips1-2* allele (Flag605F08; Ws background), the T-DNA was inserted in the 4th exon (Figure S1A). Homozygous mutants were screened by PCR; as shown on Figure S1B, both identified alleles are knock-outs for *AtIPS1*. The *Arabidopsis* genome contains three genes encoding myo-inositol phosphate synthases. We also obtained mutant lines for *atips2* and *atips3*, (see Figure S1 for details); *atips2* mutants did not display spontaneous lesion formation, while disruption of *AtIPS3* appeared to be lethal, because we could never recover homozygous plants (data not shown); we therefore focused our study on the *atips1* mutant.

**Figure 2:**
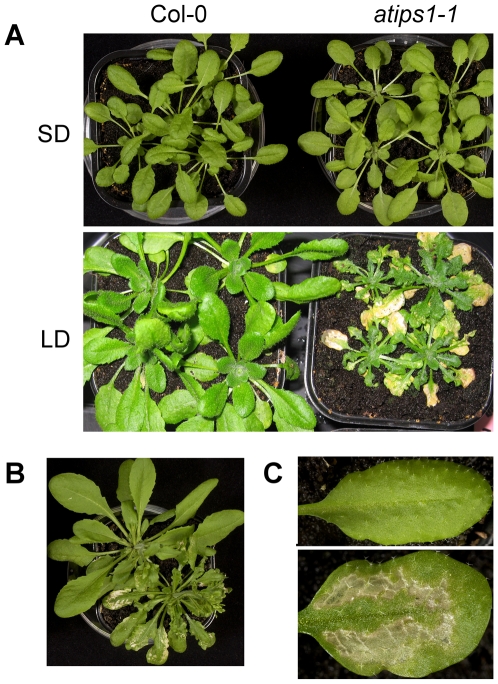
Disruption of *AtIPS1* induces spontaneous lesion formation under long day conditions. (A) Top: Wild-type (left) and *atips1-1* (right) plants grown under SD (45 µE/m^2^/s); Bottom: Wild-type (left) and *atips1-1* (right) plants grown under SD (45 µE/m^2^/s) and transferred under LD (45 µE/m^2^/s) for 4 weeks. (B) Phenotype of Ws (top left) and the *atips1-2* mutant (bottom right). (C) Close-up on a leaf from the wild-type (top) and *atips1-1* (bottom).

Most experiments were performed on both alleles, and unless otherwise specified, identical results were obtained. *In vitro* grown *atips1* mutants displayed several phenotypic alterations even when grown under short days (SD: 8 h day/16 h night, 45 µE/m^2^/s). First, the organisation of the root cap was strongly modified: cells were smaller, aberrant division planes could be observed and the distribution of amyloplasts in the columella cells was altered (data not shown). Second, seedlings were shorter (data not shown). To determine whether this was due to defects in cell division or cell elongation, epidermal hypocotyl cells were counted. We found a significantly lower cell number in *atips1-1* (x = 19.7) compared to Col-0 (x = 26.2) (t_14_ = 0,6), indicating that cell proliferation is reduced during *atips1* embryogenesis since cortical and epidermal cell divisions are absent during the elongation of *Arabidopsis* hypocotyl seedlings [22]. Finally, *atips1* cotyledons appeared deformed and displayed abnormalities in vein formation (Figure S2). However, these developmental defects were observed only on seedlings, and when plants were transferred to soil and grown under SD, they became indistinguishable from the wild-type. The most striking aspect of *atips1* mutants' phenotype is thus the spontaneous lesion formation observed when plants were transferred under long days (LD: 16 h day/8 h night 45 µE/m^2^/s) (Figure 2); we therefore focused our study on this aspect of the phenotype.

### Lesion formation in *atips1* is modulated by light intensity and the developmental stage

**Figure 3:**
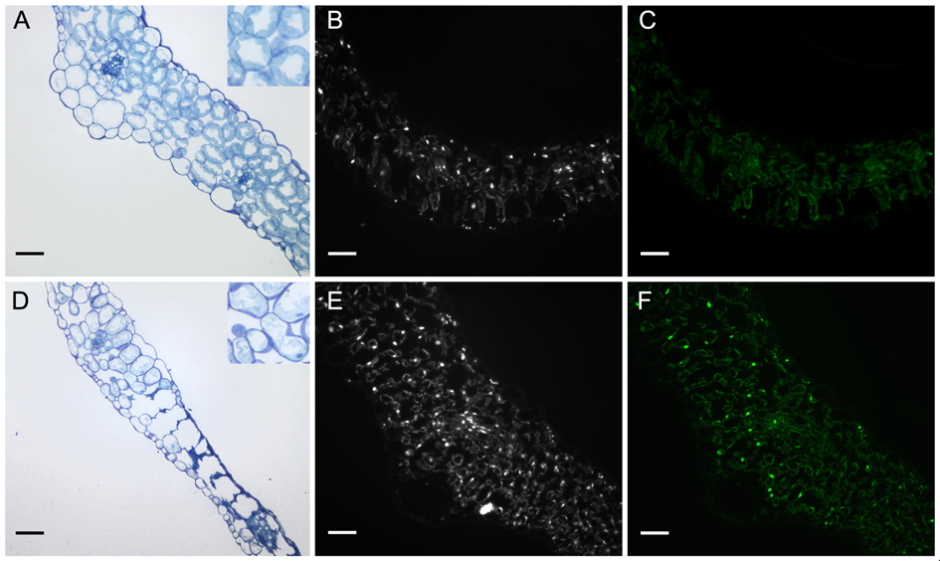
Microscopic analysis of lesions in *atips1* mutants. (A, D) Leaf sections from wild-type (A) and *atips1-1* (D) plants grown under SD (45 µE/m^2^/s) and transferred under LD (45 µE/m^2^/s) for 4 days. (B, E) Cross-section of WT (B) and *atips1-1* (E) leaves stained with DAPI. (C, F) Cross-section of WT (C) and *atips1-1* (F) stained by TUNEL. Scale bar  = 50 µm for all panels. Inserts on panels A and D are two-fold magnifications of the image.

As stated above, when grown under SD, mature *atips1-1* mutants were indistinguishable from the wild-type (Figure 2A top). However, when these plants were transferred to LD to induce flowering, we observed lesion formation on mature leaves (Figure 2A bottom). The *atips1-2* mutant displayed the same phenotype (Figure 2B). Lesion formation could be attributed to *AtIPS1* disruption since *atips1* mutants re-transformed with a *35S::AtIPS1* construct no longer formed lesions (data not shown). Lesions typically appeared four days after transfer and could spread until they covered the whole leaf, but on most leaves, the main vein and leaf margins remained green (Figure 2C). Mutants also displayed severe growth reduction, and newly formed leaves were crumpled and serrated. To determine more precisely where lesions started we performed leaf sections (Figure 3A, D). Lesions with collapsed, organelle-free cells were observed on each side of secondary veins. Neighbouring cells contained degenerating chloroplasts, indicating that the lesions were progressively reaching the veins (Figure 3D).

In addition, growth of *atips1* was drastically reduced compared to the wild-type under LD (Figure 2A). This could stem from an inhibition of either cell proliferation or cell growth. To discriminate between these two possibilities, we measured leaf surface area and cell size of *atips1-1* mutants. In *atips1-1* subjected to long-day conditions, we observed a three fold reduction of leaf surface area (Figure S3B, D). Leaf cells were slightly smaller than that of the wild-type, since we observed an increase in the number of cells per mm^2^ (Figure S3A, C). However, this moderate change in cell size cannot account for the reduction of leaf surface area. We therefore concluded that the leaves of the mutants contain fewer cells, pointing to an inhibition of cell division.

We next wondered whether lesion formation and growth inhibition were triggered by photoperiod or light intensity. To test this, *atips1-1* mutants were cultivated under high-light and SD conditions (8 h day/16 h night 225 µE/m^2^/s). Lesion formation was observed in these conditions, demonstrating that it is triggered by the quantity of light received by the plant rather than by day length. Nevertheless, it is worth noting that under these conditions lesions appeared after one week instead of 4 days, they spread more slowly and plant growth was much less reduced (Figure S4).

The extensive lesions in *atips1-1* and *atips1-2* did not prevent flowering and seed setting, although seed production was decreased relative to the wild type (data not shown). Interestingly, the extent of cell death was dependent on the developmental stage at which plants were transferred under LD: the older the plants at the time of transfer, the fewer lesions were observed on leaves. Furthermore, although *atips1-1* and *atips1-2* had qualitatively identical phenotypes, *atips1-2* appeared more severely affected. We thought that this could be due to the fact that *atips1-2* is in the Ws background, whereas *atips1-1* is in the Col-0 background. One difference between Col-0 and Ws is that Ws flowers earlier than Col-0. To determine whether flowering time could affect *atips1* phenotype, *atips1-1* was crossed with the *gigantea-6* (*gi-6*) mutant. GIGANTEA is involved in phytochrome B-controlled signalling [23]. In *Arabidopsis*, the loss of *GIGANTEA* causes a delay in flowering under LD but has negligible effect in SD [24], [25]. Since *gi-6* was in the Lansberg *erecta* (L*er*) background and *atips1-1* mutations in a Col-0 background, a backcross of *atips1-1* to the wild-type L*er* was performed as a control. Plants from the second generation clearly showed that cell death occurred in the L*er*/Col-0 background whereas it was drastically reduced in *atips1-1/gi-6* double*-*mutants (Figure S5).

Taken together, our results show that *atips1* is a conditional lesion mimic mutant (LMM): it displays spontaneous lesion formation and severe growth inhibition in a light and development-dependent manner.

### Lesion formation in *atips1* is due to salicylic acid-dependent PCD

The LMM phenotype of *atips1* could be due to necrosis or PCD. In the latter case, DNA fragmentation would be observed in the mutant and lesion formation would be regulated by phytohormones, namely SA.

DNA fragmentation in *atips1* mutants was investigated with TUNEL assays on leaf sections from wild-type and mutant plants. Leaves were harvested on plants one week after their transfer to LD. Mature leaves with little or no lesions were harvested on *atips1-1* mutants. After the TUNEL reaction, leaf sections were stained with DAPI (Figure 3B, E) to make sure that the nuclei of TUNEL negative cells were visible. As shown on Figure 3C, only few TUNEL positive nuclei could be detected in the wild-type. By contrast, strong TUNEL labelling was observed in *atips1* leaves (Figure 3F). At later stages, we observed chromatin condensation in *atips1* nuclei (data not shown). These results suggest that the observed lesions are due to PCD. To confirm this, we asked whether their formation in *atips1-1* is a regulated process.

**Figure 4:**
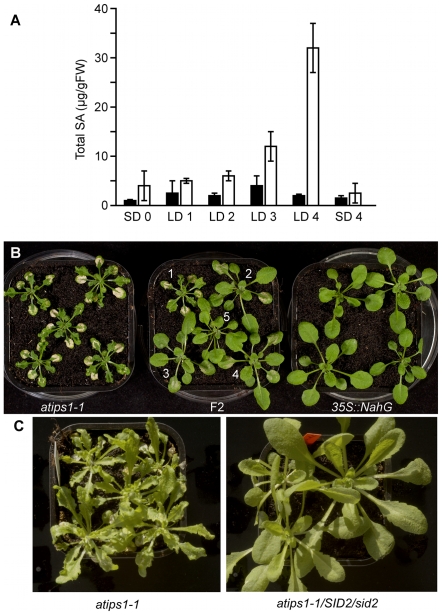
Lesion formation is dependent on salicylic acid accumulation. (A) Total SA content of wild-type (black bars) and *atips1-1* (white bars). Plants were grown under SD (45 µE/m^2^/s) for one month and transferred under LD (45 µE/m^2^/s) for one, two, three or four days or maintained under SD for four days. Total SA was quantified daily after transfer. (B) Phenotype of *atips1-1* (left), *35S::NahG* (right) and F2 plants *atips1-1x35S::NahG* (middle) two weeks after transfer under LD (45 µE/m^2^/s) conditions. In the F2 progeny all plants shown are homozygous for the *atips1-1* mutation. Plant 1 did not inherit the *35S::NahG* construct. Plants 2–5 contain the *35S::NahG* construct but probably express various levels of the protein resulting in partial (plants 2–4) to complete (plant 5) rescue of the phenotype. (C) Phenotype of *atips1-1* (left) and *atips1-1*/*SID2*/*sid2-1* mutants. Inactivation of only one copy of the *ISC1* gene is sufficient to prevent lesion formation in the *atips1-1* background.

About 40 LMM mutants have been described in *Arabidopsis*. In many but not all of them, SA production has been shown to be required for lesion formation (reviewed in [5]). Similarly to these mutants, SA content was increased in *atips1-1* prior to lesion formation. As shown on Figure 4A, total SA content of wild-type plants remained constant at around 3 µg/g FW for 4 days after transfer from SD to LD conditions. By contrast, in *atips1-1* a significant increase in total SA content could be observed 2 to 3 days after transfer and before lesion appearance. Four days after transfer, total SA was ten times higher in *atips1* than in the wild-type.

To determine whether lesion formation was dependent on this SA accumulation, *atips1-1* was crossed with a *35S::NahG* transgenic line, which cannot accumulate SA [26]. In the F2 progeny, we observed partial to complete suppression of lesion formation upon transfer to LD (Figure 4B), indicating that SA accumulation is required for lesion formation in the mutant. The variability observed in *atips1-1/35S::NahG* plants is likely to result from different expression levels of the *35S::NahG* construct. It has been shown previously that *35S::NahG* plants presented SA independent phenotypes (e.g. [27]). To confirm that lesion formation required SA production in *atips1-1*, we crossed this mutant with the *sid2-1* mutant [28], defective for isochorismate synthase (encoded by the *ICS1* gene), a chloroplastic enzyme involved in SA biosynthesis [29]. As shown on Figure 4C, *atips1-1/SID2/sid2* double mutants did not display lesion formation. It is worth noting that lesion formation was suppressed in plants homozygous for the *atips1-1* mutation but heterozygous for the *sid2-1* mutation suggesting that reduced SA biosynthesis is sufficient to abolish lesion formation, indeed, SA accumulation was slightly reduced in *SID2*/*sid2* plants [28]. To confirm this result, we analysed the progeny of a *atips1-1/SID2/sid2* plant. We found that 3/4 of the analysed plants (n = 76) did not display lesion formation, and confirmed by PCR that lesion formation was abolished both in *sid2-1* mutants and in plants heterozygous for the *sid2-1* mutation. This result is surprising since we observed only partial complementation in some *35S::NahG* plants. However, as stated above *35S::NahG* plants display SA independent phenotypes. JA and SA may function antagonistically to regulate PCD [30]. We therefore crossed the *atips1-1* mutant with the *aos* (allene oxide synthase) mutant which is deficient for JA biosynthesis [31]. Lesion formation was enhanced in the *atips1-1*/*aos* double mutant (data not shown), further confirming that phytohormones modulate lesion formation in *atips1*.

Taken together our results strongly suggest that lesions are due to PCD. We conclude that PCD and growth reduction in the mutant depend mainly on SA production.

### PCD in *atips1* is caused by drastic reduction of myo-inositol and galactinol accumulation

**Figure 5:**
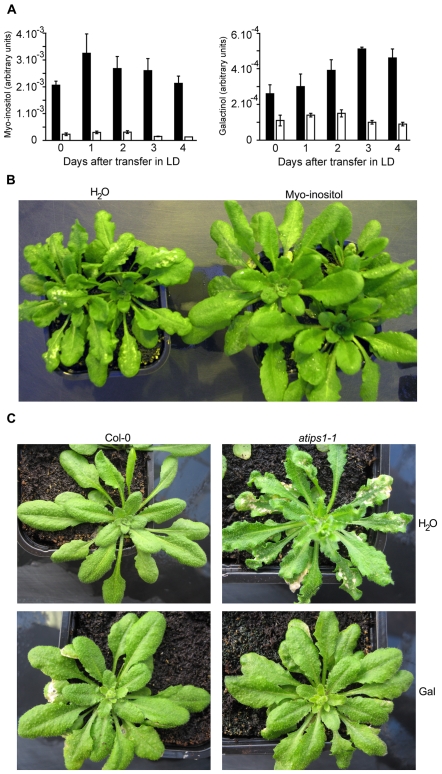
Myo-inositol and galactinol accumulation is drastically reduced in *atips1* mutants. (A) Myo-inositol (left) and galactinol (right) were quantified by GC-TOF-MS in WT (black bars) and *atips1-1* (white bars) plants grown under SD, and daily for 4 days after transfer under LD conditions. (B) Lesion formation can be abolished by treating *atips1-1* with myo-inositol. (C) Lesion formation can be abolished by treating *atips1-1* with galactinol (gal). For B and C, plants were grown for one month under SD, and transferred under LD for two weeks. They were treated daily with a myo-inositol (100 mg/mL) or a galactinol (10 mM) solution or water. Lesion formation was still obvious in plants treated with water, but not in plants fed with myo-inositol (100 mg/mL) or galactinol (10 mM). Galactinol treatment slightly affected wild-type plants development.

IPS is an enzyme involved in primary metabolism. In potato, knock-down of IPS via an anti-sense approach resulted in a drastic reduction of myo-inositol levels, but also in an increase in glucose, sucrose and starch accumulation, suggesting an alteration of the carbon primary metabolism [18]. To determine how cellular metabolism was affected in *atips1* mutants, we performed metabolomic analyses on the mutant after transfer to LD using the GC-TOF-MS approach described by Noctor *et al.* [32]. To follow potential metabolic changes after transfer to LD, leaves were harvested daily, for four days after transfer. Detected metabolites are listed in Table S1. Surprisingly, *atips1* differed from the wild-type only for the content of myo-inositol and galactinol, a compound synthesised by conjugation of UDP-galactose and myo-inositol [33] (Figure 5A); we did not observe significant changes in glucose or sucrose accumulation. Conversely, *atips1* mutants did not accumulate more starch than the wild-type and ^31^P-RMN analyses did not reveal any modifications in pools of Calvin cycle intermediates such as ribulose-1,5-bisphosphate (data not shown).

To test whether the induction of PCD could be attributed to the reduced myo-inositol or galactinol accumulation, *atips1* mutants were transferred to LD, and either sprayed with a 100 mg/mL myo-inositol solution or brushed with a 10 mM galactinol solution. As shown on Figure 5B–C, water-treated plants still displayed lesion formation, whereas PCD was clearly reduced in myo-inositol- and galactinol-treated plants. Recently, galactinol has been proposed to function as a ROS scavenger under stress conditions such as chilling or high-irradiance [33]. We therefore tested whether reduction of galactinol contents resulted in enhanced ROS sensitivity in *atips1* mutants, with wild-type and *atips1* plants cultivated under SD on MS for 12 days and then transferred to MS medium supplemented with various drugs known to induce oxidative stress (see Figure S6 for details). Surprisingly, *atips1* did not show enhanced susceptibility to oxidative stress, but seemed instead more tolerant than the wild-type to norflurazon.

**Figure 6:**
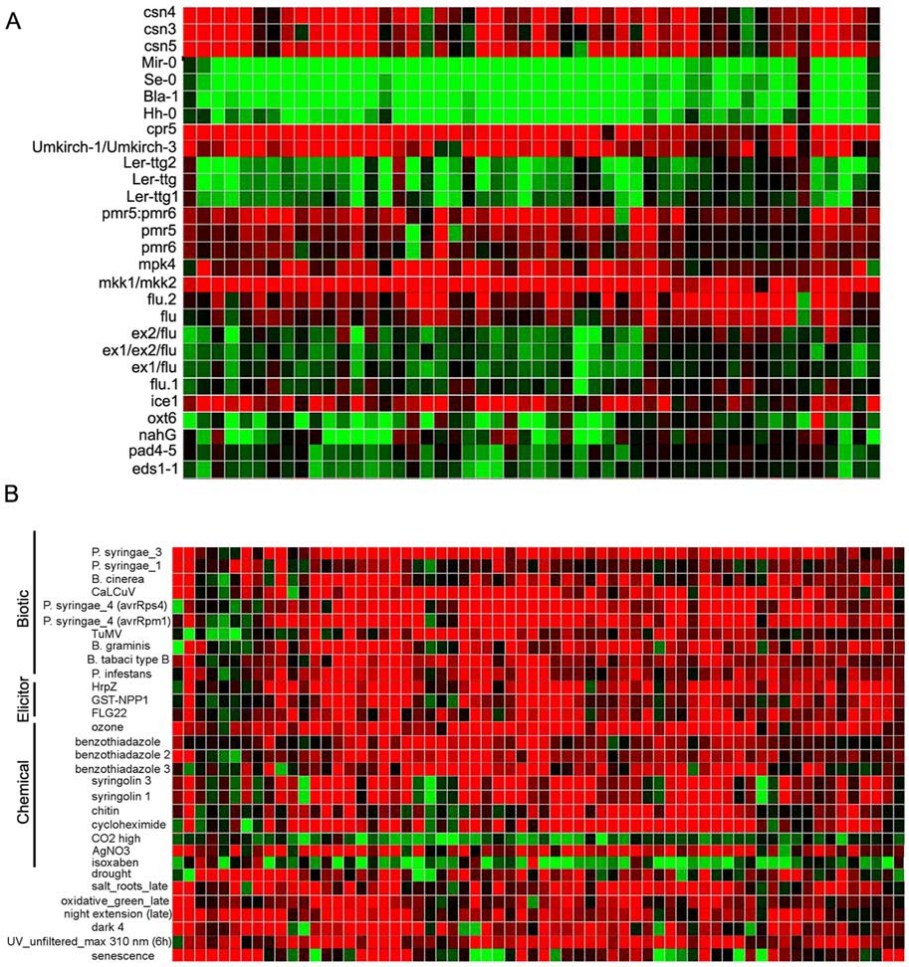
The transcriptome of *atips1-1* is similar to that of several LMM mutants or plants infected by pathogens. Hierarchical clustering was performed using 150 transcripts across the different SD/LD conditions. Each vertical line displays the expression data for one gene. List of genetic backgrounds or treatment are displayed horizontally. Red and green indicate up- and down-regulation in mutants (A) or treated plants (B) compared to wild-type or untreated plants, respectively. Intensity of the colours is proportional to the absolute value of the fold difference. Images presented here correspond to a representative region of the global image which was too wide to be reproduced integrally.

Taken together, these results suggest that spontaneous cell death in *atips1* is due to a decrease in myo-inositol and galactinol accumulation. However, these molecules, and especially myo-inositol can take part in various cellular processes, and further analysis was therefore required to understand the cellular processes triggering PCD in *atips1*.

### Transriptome analysis of *atips1-1*

To explore the molecular processes underlying the cell death phenotype of *atips1*, and to determine whether this mutant differed from the wild-type under permissive conditions, its transcriptome was analyzed using CATMA whole genome micro-arrays as described in the methods section. Wild-type and mutant plants were grown under SD (45 µE/m^2^/s) for 3 weeks and then one part of the plants was transferred to LD conditions (45 µE/m^2^/s) to induce lesion formation. Three comparisons were carried-out: Col-0 vs *atips1-1* grown under SD (Table S2), Col-0 vs *atips1-1* four days after transfer under LD (Table S2) and *atips1-1* grown under SD vs *atips1-1* four days after transfer under LD (Table S3). Two independent RNA extractions and micro-array experiments were performed from two independent biological replicates. Table S2 summarizes all nuclear genes differentially expressed between *atips1-1* and Col-0 under SD or LD conditions. *AtIPS1* expression was clearly down regulated in all the conditions. Interestingly, we found that only 271 genes were differentially expressed in *atips1-1* under SD (223 down-regulated and 48 up-regulated), while under LD conditions 1856 genes were either up (1032) or down-regulated (824). Furthermore, almost all down-regulated genes under SD were up-regulated under LD (175 out of 223) and reciprocally, most up-regulated genes under SD became down-regulated under LD (36 out of 48). Among the 1032 up-regulated genes under LD, 72 had putative function in biotic stress response such as *PR5*, *AIGI*, *BGL2, EDS1* or *PAD4* (Table S4) and 52 were potentially involved in oxidative stress response (Table S5). These observations are consistent with the formation of HR-like lesions. CATMA micro-arrays include 277 probes covering the chloroplastic genome, among which 111 displayed reduced signals in *atips1-1* under LD (Table S6). Furthermore, 251 out of the 1311 (i.e. 19%) genes encoding plastid targeted protein according to the CATMA array annotation were down-regulated in *atips1-1* under LD. Only 3.5% of the genes represented on the array were down-regulated in *atips1-1*: genes encoding plastidial proteins are therefore significantly more affected than the others in the mutant. Taken together, these results reveal that chloroplastic function is severely impaired in *atips1-1* mutants under restrictive conditions.

To validate the micro-array analysis, expression of *ZF14* and *WRKY53* were monitored by qRT-PCR. We chose these two genes because they showed high and moderate up-regulation under LD respectively. As shown on Figure S7, qRT-PCR assays were consistent with the micro-array results.

We next compared the *atips1-1* transcriptome to transcriptome data available from public library obtained either on wild-type plants treated with various stimuli or on mutants, using the Genevestigator software [34]. This comparison was performed using genes that were up-regulated more than 2.8 fold under LD (i.e. 150 genes). As shown on Figure 6A, we could define two groups of genotypes. Plants from the first group have a transcriptome profile opposite to that of *atips1-1*: genes that are up-regulated in *atips1-1* are down-regulated in these plants. This group includes several wild-type plants of different ecotypes such as Bla-1, Se-0 or Mir-0, whose hybrids displayed an increase in functional immune responses [35]. Interestingly, Mir-0 is extremely late flowering compared to Col-0. Plants from the second group over-express the same genes as *atips1-1*. This includes mutants forming spontaneous lesions such as *cpr5* [36] or plants displaying hybrid necrosis [35], and mutants which constitutively express SA induced genes such as *mkk1*/*mkk2* or *mpk4* [37], [38]. Furthermore, comparison of *atips1-1* transcriptome with that of wild-type plants subjected to various stimuli revealed that up-regulated genes in *atips1-1* under restrictive conditions are induced by various types of stress such as salt or drought, pathogen attacks or ozone treatment (Figure 6B). Like pathogen attacks, ozone treatment induces an oxidative burst and HR-like lesion formation [4].

### Disruption of *AtIPS1* affects pathogen resistance

**Figure 7:**
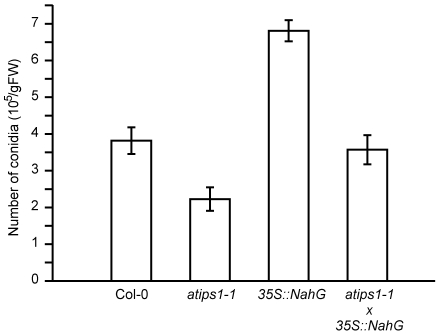
Characterization of *atips1-1* innate immunity. Growth of virulent *Hyaloperonospora parasitica* Noco2 on Col-0, *atips1-1, 35S::NahG,* and *atips1-1/35S::NahG* plants was estimated by conidia numeration 6 days after inoculation as described in the methods section.

As described above, the transcript profile of *atips1-1* was very similar to that of plants subjected to biotic stress or LMM. Several LMM display enhanced pathogen resistance, either to virulent or avirulent strains (reviewed in [5]). These results prompted us to test *atips1* tolerance to biotic stress. Murphy *et al*. tested *atips1-1* susceptibility to both virulent and avirulent strains of *Pseudomonas syringae* and did not report any difference with the wild-type [20], we therefore tested an oomycete pathogen: *Hyaloperonospora arabidopsis* (formerly *H. parasitica*). Col-0 and *atips1-1* mutants were infected with the virulent (Noco2) isolate of *H. arabidopsis*. This pathogen causes downy mildew disease on wild populations of *Arabidopsis* and is a destructive pathogen of cultivated Brassicaceae. This isolate is virulent on the Col-0 ecotype, but not on the Ws ecotype [39]; tests were therefore performed on the *atips1-1* line under SD conditions. As shown on Figure 7, a two fold reduction was observed in the number of conidia formed on *atips1-1* mutants compared to the wild-type. By contrast, *35S::NahG* plants were more susceptible to this pathogen than the wild-type as described by Delaney *et al.* [40], and *atips1*/*35S::NahG* plants were comparable to the wild-type. Taken together, these results suggest that basal resistance is enhanced in *atips1*, and that this improved resistance is dependent on SA accumulation.

## Discussion

A yeast two-hybrid screening aimed at identifying protein partners for the PCNA-binding protein ATXR5 [7] drew our interest to AtIPS1 and to the role of MI metabolism in the control of cell proliferation and PCD in *Arabidopsis*. To elucidate these questions we characterised *Arabidopsis atips1* mutants.

### *Arabidopsis* IPS have non-redundant functions

In their work on potato plants affected in *StIPS* expression, Keller *et al* previously reported that reduction of MI biosynthesis resulted in a variety of morphological and physiological changes, and concluded that the various phenotypes were probably due to changes in more than one compound [18]. Interestingly, although there are three isoforms of IPS in *Arabidopsis* sharing more than 90% identity in their amino-acid sequence, the three mutants have contrasting phenotypes: we found that disruption of *AtIPS1* affects several aspects of seedling growth and development and results in spontaneous lesion formation on leaves. Plants mutated for AtIPS2 (line *atips2*) are identical to the wild-type with respect to growth and development but seem to be affected for pathogen resistance [20], while *AtIPS3* appears to be essential for embryo development since homozygous *atips3* mutants cannot be obtained. Surprisingly, Murphy *et al* did not report spontaneous lesion formation in their analysis of the *atips1-1* mutant. This discrepancy is difficult to explain because the growth conditions described in their work are very similar to those used in our study. Furthermore, lesion formation can be attributed to disruption of *AtIPS1* since two independent alleles exhibit this phenotype and also because it could be complemented both by introducing the *AtIPS1* cDNA and when supplementing the plants with MI.

Thus, AtIPS1, 2 and 3 seem to have non-redundant functions in *Arabidopsis*. It has been suggested that different cellular pools of MI may fulfil distinct functions [20]. In support to this hypothesis, AtIPS1, 2 and 3 are predicted to be targeted to distinct cellular compartment [41]. Interestingly, AtIPS1 is the only isoform harbouring a putative nuclear localisation signal [41]. This is in agreement with its ability to enter the nucleus (Figure 1B–C) and to bind ATXR5/6, and may account for the involvement of this particular IPS in the control of PCD as will be discussed later on.

### *atips1* is a LMM displaying enhanced basal defence

The most striking feature of *atips1* mutants is the spontaneous lesion formation on leaves. Although down-regulation of *StIPS* has been reported to induce early senescence [18], we think that lesion formation in *atips1* is more related to HR than to senescence. Indeed, expression of the senescence marker *SAG12* [42] remains unchanged in *atips1-1* after transfer under restrictive conditions. Furthermore, the transcriptome of *atips1-1* mutant is highly similar to that of wild-type plants facing pathogen attacks or treated with ozone, a method commonly used to simulate HR, but not to that of senescing plants (Figure 6B). Therefore *atips1* should be regarded as a LMM. LMM have been classified into two groups: initiation mutants that form localized cell death spots of determinate size and propagation mutants which are unable to control the rate and extent of the lesions [5]. According to its phenotype, *atips1* is a propagation mutant, like *acd1*, *acd2* or *lsd1* [5]. Indeed, the phenotype of *atips1* is very similar to that of *lsd1*, a mutant that forms lesions in a light-dependent manner [43].

Similarly to several LMM, we found that lesion formation in *atips1* requires SA accumulation. Since *atips1* mutants constitutively express genes involved in pathogen response similarly to mutants that show increased pathogen resistance such as *cpr5* [36], one could expect that *atips1* should be more resistant than wild-type to pathogen attacks. Murphy *et al.* found that *atips1* resistance to the avirulent pathogen *Pseudomonas syringae* harbouring the avirulent gene *AvrB* and to viruses was similar to that of the wild-type, indicating that HR is not modified in this mutant [20]. By contrast, we found that *atips1* is more resistant than wild-type to the Noco2 isolate of *H. parasitica*, suggesting that basal defence is enhanced in the mutant. This could be attributed to an increased production of SA in response to pathogen inoculation, since *atips1-1/35S::NahG* plants were as susceptible as the wild-type to this pathogen. However, because the phenotypes of *atips1-1* and *35S::NahG* plants are additive, we cannot rule out the possibility that *AtIPS1* and SA may affect plant resistance to *H. parasitica* via independent mechanisms.

### Lesion formation in *atips1* is developmentally regulated

Our results suggest that lesion formation in *atips1* is affected both by the amount of light received by the plants and by day-length. Indeed, *atips1* mutants displayed more severe lesion formation and growth reduction when grown under 16 h light at 45 µE/m^2^/s than when grown under 8 h light at 225 µE/m^2^/s, even though the total amount of light received per day was much higher under the latter conditions. In addition, the severity of symptoms of *atips1* mutants in restrictive conditions was much reduced if plants were kept for several weeks under permissive conditions. These observations suggest the existence of cross-talks between developmental signals and the cellular mechanisms responsible for lesion formation in *atips1* mutants. In agreement with this hypothesis, we observed that genes up-regulated in *atips1-1* under restrictive conditions are down-regulated in the Mir-0 ecotype that flowers over a month later than Col-0 in our growth conditions. Furthermore, we observed that lesion formation was drastically reduced when the *atips1* mutation was introduced in the *gi-6* background that strongly delays flowering. Although we cannot rule out that the latter result could be caused by the enhanced tolerance of *gi* to oxidative stress, due to increased ascorbate peroxidase activity [44], [45], our results are in agreement with the assumption that oxidative stress tolerance and longevity are linked in *Arabidopsis* [45]. Recently, Achard *et al* demonstrated that DELLA proteins can regulate both growth and survival under stress conditions, providing putative molecular basis for this interplay between plant development and stress response [46].

### The onset of PCD in *atips1* may involve signals coming from chloroplasts

The observation of TUNEL-positive cells prior to lesion formation strongly supports the view that *AtIPS1* is required to repress a PCD programme under given environmental conditions. In fact, DNA fragmentation is believed to be a marker of PCD [3]. Furthermore, we showed that lesion formation is mainly dependent on SA accumulation, demonstrating that it is not due to necrosis but to a regulated cellular process. In *atips1*, the onset of PCD appears to be triggered when plants are exposed to a high irradiance (see above), and many up-regulated genes under restrictive conditions are involved in oxidative stress response, pointing to a potential role of ROS production in chloroplasts. In both animal and plant cells, mitochondria are cellular executioners of PCD. Their central role involves integrating stress and/or PCD signals that ultimately cause the release of mitochondrial molecules which in turn trigger cell-death cascades [47]. In plant cells, ROS can be generated in several cellular compartments, including chloroplasts. Recently, evidence has been provided for a role of chloroplasts in HR-like cell death in tobacco [48]. Likewise the LSD1 protein probably functions as an integrator of chloroplast-derived redox signals to regulate programmed cell death in response to excess light [49]. Possibly then, PCD in *atips1* mutants could be induced by increased ROS production in chloroplasts. In support to this hypothesis, we observed that lesion formation is abolished in *atips1* mutants re-transformed with a construct encompassing an artificial micro-RNA (a-miRNA) targeting *GUN4* ([50], Figure S8). Originally, *gun* mutants were isolated for their deficiency in nuclear gene repression following chloroplastic damage [51], and are therefore assumed to be affected in chloroplast to nucleus signalling. However, GUN2-5 proteins were all found to be involved in tetrapyrrole biosynthesis; as a result, down-regulation of *GUN4* via amiRNA led to reduced chlorophyll accumulation. Interestingly, Ishikawa reported that disruption of tetrapyrrole biosynthesis suppressed lesion formation in *len1*, another LMM mutant [52].

That said, the observation that *atips1-1* mutants are not more sensitive than the wild-type to oxidative stress may appear conflicting with the hypothesis that PCD in the mutant could be triggered by ROS production in chloroplasts. Interestingly, microarray analysis revealed that up-regulated genes under permissive conditions become down-regulated after transfer to restrictive conditions. Reciprocally, down-regulated genes under SD were up-regulated under LD. We propose that *atips1-1* mutants grown under permissive conditions may have become acclimated to a certain constitutive level of stress, allowing them to better face moderate oxidative stress than wild-type. Upon transfer to restrictive conditions, oxidative stress in the mutant may reach a threshold above which PCD would be triggered. Lesion formation in *atips1* could hence be triggered by chloroplastic signals, while the role of MI or MI derivatives in the cellular response to these signals remains to be established.

### What is the link between MI metabolism and cell cycle or PCD regulation?

The cellular mechanisms underlying lesion formation and the increase in pathogen resistance are not clear. They could be due in part to modifications in inositol signalling. Indeed, in animal cells, PI3K and PKB are major regulators of PCD by blocking pro-apoptotic pathways (for review see [53]). To date, the role of this signalling pathway in the control of plant PCD has been little documented, but a reduction in the cellular content of MI may affect PI3K-dependent inhibition of PCD in *atips1*. In addition, Ortega *et al.* reported that IP_3_ production played a role in HR response of lemon seedlings against *Alternaria alternata* [54]. Alternatively, PCD induction in *atips1* may be due to alterations in sphingolipid metabolism as shown previously for the *acd5* and *acd11* mutants [55], [56]. Along the same line, Wang *et al.* recently demonstrated that inositol-phosphoceramides were involved in the regulation of PCD [57]: *atips1* mutants may be affected for inositol-phosphoceramide biosynthesis, and thus fail to repress PCD. Hence, spontaneous PCD in *atips1* may result from the alteration of various inositol-related signalling pathways. In agreement with such a hypothesis, we found that spraying the plants with inositol could suppress lesion formation, indicating that inositol production, and not the AtIPS1 protein itself, is required to prevent lesion formation. Lesion formation in *atips1* could also be due to the requirement of MI as a precursor for several molecules involved in stress tolerance. Indeed, *cat2* mutants which are compromised in ROS detoxification display spontaneous lesion formation when grown under LD [58]. In addition, galactinol treatment also suppressed lesion formation in *atips1*, suggesting that it could be due to enhanced oxidative stress, since galactinol has been proposed to act as a scavenger of ROS [33]. However, *atips1* did not show enhanced susceptibility to oxidative stress: galactinol may therefore fulfil other functions such as signalling in plant cells as suggested by Kim *et al.* [59]. Moreover, we cannot rule out the possibility that the rescue of the mutant phenotype by galactinol treatment could be due to MI production via galactinol degradation or raffinose biosynthesis.

Finally, cell division is drastically reduced in *atips1* mutants under restrictive conditions as well as in the hypocotyl of *atips1* embryos. Although this inhibition of cell division could be due to SA accumulation, AtIPS1 may be involved in cell cycle regulation. Indeed AtIPS1 interacts with the PCNA-binding proteins ATXR5/6 and expression of *AtIPS1*, like that of *AtPCNA* and *ATXR6* [7] is regulated by E2F transcription factors [60]. Further work shall be needed to determine whether this role is direct or indirect and to elucidate the molecular processes involved.

## Materials and Methods

### AGI numbers of genes mentioned in this study and mutant lines

*AtIPS1* and *AtIPS2* correspond to loci At4g39800 and At2g22240 respectively [20]; and *AtIPS3* corresponds to the At5g10170 locus. AGI numbers for *ATXR5* and *ATXR6* are At5g09790 and At5g24330 respectively. Mutant lines used in this study were SALK_023626, and Flag605F08 for *AtIPS1*, SALK_101349 for *AtIPS2* and SALK_071284 for *AtIPS3*.

### Plant growth

Unless otherwise specified in the text, plant growth conditions were as follows. Plants were sown *in vitro* on 0,5× MS medium (Basal Salt Mixture, Duchefa) and grown in a 12 h day growth chamber. After two weeks, plants were transferred to soil under SD conditions (8 h day, 16 h night, 21°C, 45 µE/m^2^/s) for at least one week. Plants were subsequently transferred to LD conditions (16 h day, 8 h night, 21°C, 45 µE/m^2^/s). Mutant lines were obtained from the SALK [61], and the Versailles mutant collections.

### Genotyping of the mutants

To extract genomic DNA, leaves from the mutant were ground with metal beads in 400 µL of CTAB buffer (200 mM Tris pH 7.5, 250 mM NaCl, 25 mM EDTA, 0.5% w/v SDS). After 30 min incubation at 60°C, 400 µL of chloroform were added to each sample. After centrifugation (10 min, 21000 g), the aqueous phase was recovered and DNA was precipitated by addition of 300 µL isopropanol. Samples were centrifuged (10 min 21000 g), and the DNA pellet was washed with 70° ethanol, dried and resuspended in 100 µL water. 2 µL of DNA solution were used for each PCR reaction. Homozygous plants were screened for by PCR. The position of the primers is indicated on Figure S1, primer sequence will be provided upon request.

### Constructs and Nucleic Acids Manipulations

Standard nucleic acid manipulations were performed according to [62]. For complementation of the *atips1* mutant, we cloned *AtIPS1* cDNA downstream of a 35S promoter. The full length cDNA encoding *AtIPS1* was cloned between the BamHI and XhoI sites of the Gateway compatible pEntr1A vector (Invitrogen). After sequencing, the cDNA was introduced into the pGreen0229 vector using the LR clonase (Invitrogen) according to manufacturer's instructions. The 35S promoter was subsequently replaced by the putative *AtIPS1* promoter (1000 bp upstream the initiation codon), and the resulting construct was used for transient expression assays.

For RNA extraction, biological samples were harvested, immediately frozen in liquid nitrogen and ground with 2.4 mm diameter metal beads at low temperature with a Qiagen Tissuelyser (30 Hz, 1 min). Total RNA were subsequently extracted with Tri Reagent^®^ (Sigma-Aldrich, Lyon, France) according to the manufacturer's instructions. RNA gel blot analysis were performed as described in [63]. The probe for *AtIPS1* consists of the full-length cDNA.

For reverse transcription and real-time quantitative PCR experiments, total RNAs were extracted from leaves of *Arabidopsis* plantlets using the NucleoSpin RNA Plant kit (Macherey-Nagel) including DNAse treatment. 2 µg of each sample were reverse transcribed with 25 ng/µL oligo-dT primer, 3 mM MgCl_2_, 0.5 mM dNTP and 1 µL of ImProm-II™ Reverse Transcriptase (Promega, Charbonnière, France) in a total volume of 20 µL.

1/30^th^ of the synthesized cDNA was mixed with 100 nM of each primer and LightCycler^®^ 480 Sybr Green I master mix (Roche Applied Science) for real time quantitative PCR. Products were amplified and fluorescent signals acquired with a LightCycler^®^ 480 detection system. The specificity of amplification products was determined by melting curves. Exor4 relative quantification software module (Roche Applied Science) calculates relative expression level of the selected genes with algorithms based on ΔΔCt method. Data were from duplicates of at least two independent experiments.

AtACT2 was used as internal control for signals normalization.

### Light and SEM Microscopy

Plant tissues were fixed in ethanol/acetic acid (3∶1 v/v), and incubated in chloralhydrate (4 g in 1 mL water + 1 mL glycerol) over-night at room temperature. Images were taken with a Nikon Coolpix 990 digital camera mounted on a Leica DM R microscope.

For scanning electron microscope (Hitachi S-3000) analysis, samples were slowly frozen at −18°C under partial vacuum on the Peltier stage before observation under the ESSED mode. Cell area were measured with the Image J software as described in [64].

Protoplast transformation and confocal microscopy on transiently transformed BY-2 cells were performed as described in [65].

### TUNEL assay

For TUNEL assay, leaves from *atips1-1* and wild-type plants were fixed in paraformalhedyde (4% in PBS, pH 7,4) under vacuum at room temperature for 1 h, and kept in fresh paraformalhedyde at 4°C overnight. After PBS washing, samples were embedded in paraplast. 8 µm sections were placed on a glass plate and paraplast was removed. Samples were washed with water and incubated in freshly prepared permeabilization solution (Natrium citrate 0.1%, triton 0.1%) for 8 min. After PBS washing, the TUNEL reaction was performed using the TUNEL in situ cell death detection kit-fluorescein (Roche applied science) according to manufacturer's instructions. To reduce unspecific signal, the reaction buffer was diluted two times in dilution buffer (Roche). After PBS washing, samples were mounted in Vectashield with DAPI (Vector). Samples were observed using an epifluorescence microscope (Axioskop, Zeiss). Excitation and emission filters were as follows: for DAPI, excitation was between 353 and 377 nm and emission was above 397 nm while for fluorescein, excitation was between 450 and 490 nm and emission was between 515 and 565 nm. Images were acquired using a digital camera (RT SPOT, Diagnostic instrument, Inc, USA).

### Quantification of salicylic acid

Wild-type and mutant plants were grown under SD conditions for four weeks, and subsequently transferred to LD conditions. About 100 mg of leaves were harvested each day after transfer to LD conditions and used for salicylic acid quantification. Total salicylic acid (SA) was extracted and analysed as described by Baillieul *et al*. [66] with a Nova-Pak 4 µm C-18 column (150×3.9 mm, Waters corporation, Milford, U.S.A) as part of the Waters system (1525 Binary HPLC pump, 2475 Multi λ Fluorescence Detector, 2996 Photodiode Array Detector, 717 Autosampler, Waters corporation, Milford, U.S.A). Data were analysed using Empower Pro Software (Waters corporation, Milford, U.S.A). Corrections for losses were done as described previously [66], using a LS 6500 Multi-Purpose Scintillation Counter (Beckman Coulter, Fullerton, U.S.A). Data presented here are the average of the results obtained from three distinct samples of mutant and wild-type plants.

### Plant infection by *Hyaloperonospora Arabidopsis*

The oomycete pathogen *H. arabidopsis* (Noco2 isolate) [39] was maintained by transferring conidiospores weekly onto new healthy Col-0 seedlings. Conidiospores were harvested by vortexing infected seedlings in water. The conidiospore concentration was determined using a haemocytometer and adjusted to 1×10^5^ spores per ml. Ten mg of seeds of each line were sawn in 3 different plugs. Ten-day-old seedlings were sprayed to saturation with the conidiospore suspension then maintained under high humidity for 24 hours. Infected plants were kept at 20°C under SD conditions (8 h of light, 16 h of dark). 5 days after infection, plants were sprayed with water to induce sporulation and then kept for 48 hours under high humidity. Conidiospore production was evaluated 7 days after infection. All plants (cotyledons and small leaves) from separate plugs were cut and weighed. Spores were liberated by vortexing the harvested plant tissues in 10 ml of water for 10 minutes. Spores from five samples from each separate plug were counted using a haemocytometer. Samples were scored twice to ensure accuracy. The values were then converted to the number of spores per mg of fresh weight.

### Metabolomic profiling

GC-TOF-MS was performed on a LECO Pegasus III with an Agilent 6890N GC system with Agilent 683 automatic liquid sampler. The column was an RTX-5 w/integra-Guard (30 m×0.25 mm i.d. +10 m integrated guard column) (Restek, Evry, France).

Leaf samples (100 mg fresh weight) were rapidly frozen in liquid N_2_ and stored at −80°C until extraction. Each sample typically contained leaves from 3 rosettes and duplicates were analysed for each sample. Samples were ground in a mortar in liquid N_2_ then in 2×1 mL extraction medium consisting of 80% methanol containing 100 µM Ribitol as internal standard. Extracts were transferred to 2 mL eppendorf tubes, and then centrifuged at 10,000 *g* and 4°C for 15 minutes. Supernatants were transferred to fresh tubes and centrifuged again. Several aliquots of each extract (0.1 mL, 3×0.2 mL and 0.4 mL) were spin-dried under vacuum and stored at −80°C until analysis.

GC-TOF-MS analyses consisted of a single injection of one dried 0.2 ml aliquot. Methoxyamine was dissolved in pyridine at 20 mg mL^−1^ and 50 µL were added. Following vigorous mixing, samples were incubated for 90 minutes at 30°C with shaking. 80 µL MSTFA were then added, the mix was vortexed, and incubated for 30 minutes at 37°C with shaking. The derivatization mix was then incubated for 2 h at room temperature, before loading into the GC autosampler, a mix of a series of eight alkanes of chain lengths between C10 and C36 were also included.

Analyses were performed by injecting 1 µL in splitless mode with 230°C as injector temperature. Separation was performed in a helium gas-stream at 1 mL min^−1^ in constant flow mode using a temperature ramp from 80 to 330°C between 2 and 18 min followed by 6 min at 330°C. Total run time per injection was 30 min. Ionization was made by electron impact at 70eV and the MS acquisition rate was 20 spectra s-1 over the *m*/*z* range 80–500, as in [67].

Peak identity was established by comparison of the fragmentation pattern with MS publicly available databases (NIST), using a match cut-off criterion of 750/1000 and by RI using the alkane series as standards.

For GC-TOF-MS, integration of peaks was performed using LECO Pegasus software. Because automated peak integration was occasionally erroneous, integration was verified manually for each analysis.

### Transcriptome studies

The microarray analysis was performed at the Unité de Recherche en Génomique Végétale (URGV), (UMR INRA1165 – CNRS8114) using the Complete *Arabidopsis* Transcriptome MicroArray (CATMA) [68] containing 24276 gene specific tags (GSTs) from *Arabidopsis* and 384 controls. Plants were grown for one month under SD conditions (45 µE/m^2^/s) and transferred under LD condition (45 µE/m^2^/s) for 4 days. Total RNAs were extracted from Col-0 and *atips1-1* kept under SD and under LD. RNA samples from 2 independent biological replicates were used. For each biological repetition, each RNA sample was obtained by pooling fresh material from 4 different plants One dye swap (technical replicate with fluorochrome reversal) was made for each biological repetition (*i.e.* 4 hybridizations per comparison). The RT of RNA in the presence of Cy3-dUPT or Cy5-dUTP, the hybridization of labelled samples to the slides, and the scanning of the slides were performed as described in [69].

### Statistical analysis of microarray data

Experiments were designed with the statistics group of the Unité de Recherche en Génomique Végétale. Statistical analysis was based on two dye swaps (i.e. four arrays, each containing 24,576 GSTs and 384 controls) as described in [69]. Controls were used for assessing the quality of the hybridizations, but were not included in the statistical tests or the graphic representation of the results. For each array, the raw data comprised the logarithm of median feature pixel intensity at wavelengths 635 (red) and 532 nm (green). No background was subtracted. In the following description, log ratio refers to the differential expression between two conditions. It is either log2 (red/green) or log2 (green/red) according to the experimental design. Array-by-array normalization was performed to remove systematic biases. First, we excluded spots that were considered badly formed features. Then, we performed global intensity-dependent normalization using the LOESS procedure to correct the dye bias. Finally, for each block, the log ratio median calculated over the values for the entire block was subtracted from each individual log ratio value to correct print tip effects on each metablock. To determine differentially expressed genes, we performed a paired t test on the log ratios, assuming that the variance of the log ratios was the same for all genes. Spots displaying extreme variance (too small or too large) were excluded. The raw P values were adjusted by the Bonferroni method, which controls the FWER (family-wise error rate). We considered as being differentially expressed the genes with an FWER <5%.

## Supporting Information

Figure S1 Structure of AtIPS genes and position of the T-DNA insertions. (A) Gene structure of AtIPS1, AtIPS2 and AtIPS3. Exons are represented as boxes and introns as lines. The position of T-DNA insertions in the mutants used in this study is indicated for each gene. Arrows represent the primers used for identification of homozygous mutants. (B) RNA gel blot analysis of total RNA isolated from wild-type (lanes 1, 2) atips1-1 (lane 3) and atips1-2 (lane 4) EtBr: ethidium bromide.
(0.30 MB TIF)

Figure S2 Cotyledons of atips1 mutants are deformed. Five day-old plantlets of the wild-type (A) and atips1-1 mutants (B, C) were fixated in ethanol/acetic acid (3∶1 v/v) and cleared by chloralhydrate treatment. Veins form a closed network in the wild-type, while this network is open in the mutant. Arrows indicate breaks in the vein network.
(2.43 MB TIF)

Figure S3 Disruption of AtIPS1 affects cell proliferation. (A) SEM image of wild-type and atips1 leaf epidermis. Scale bar  = 100 µm. (B) Average leaf area in WT (black bars) and atips1 (white bars) plants. (C) Number of cells per surface unit in WT (black bars) and atips1 (white bars) plants for abaxial (left) and adaxial (right) epidermis. (D) Leaf size of representative atips1-1 and wild-type plants.
(6.43 MB TIF)

Figure S4 Phenotype of atips1-1 plants grown under SD and higher irradiance. Plants were grown under SD conditions. They were kept under low irradiance (45 µE/m2/s) for a month and transferred under LD at the same light intensity (A) or SD at higher irradiance (225 µE/m2/s) (B) for two weeks. Lesion formation occurred in both cases, but they spread more rapidly and plant growth was more affected in LD.
(6.89 MB TIF)

Figure S5 Lesion formation is drastically reduced in the atips1-1/gi-6 double mutant. Plants were grown under SD conditions. They were kept under low irradiance (45 µE/m2/s) for a month and transferred under LD at the same light intensity. The atips1-1 mutation induced lesion formation and growth inhibition in the Ler background. By contrast, atips1-1/gi-6 mutants form little or no lesions and grew normally, but showed delayed flowering like the gi-6 mutant (not shown).
(4.33 MB TIF)

Figure S6 Oxidative stress tolerance is not reduced in atips1 mutants. Experiments were performed under SD conditions. Wild-type (black bars) and atips1-1 (white bars) plants were cultivated on 0.5× MS for 12 days and transferred to 0.5× MS medium (MS) or 0.5× MS medium containing norflurazon (NOR) or 3-amino-1, 2, 4-triazole (3AT) and DL-buthionine-(S,R)-sulfoximine (BSO). General oxidative stress was induced by treating plants with 3AT and BSO: 3AT is an inhibitor of catalase, and therefore generates H2O2 accumulation [1], while BSO inhibits gluthation biosynthesis, thus inhibiting this ROS scavenging pathway [2]. Norflurazon is an inhibitor of carotenoid biosynthesis: plants treated with norfluorazon suffer from photooxidation of the thylakoid membrane, treatment with norflurazon therefore generates oxidative stress preferentially in chloroplasts [3]. After one week, roots were measured. We observed a two-fold reduction in root-length for wild-type plants on both media and for atips1-1 on 3AT+BSO. By contrast, NOR treatment only resulted in a 1.3 fold reduction in root growth in the mutant, suggesting that atips1-1 may be more tolerant than the wild-type to norflurazon. 1. May MJ, Leaver CJ (1993) Oxidative Stimulation of Glutathione Synthesis in Arabidopsis thaliana Suspension Cultures. Plant Physiol 103: 621–627. 2. Meister A (1995) Glutathione biosynthesis and its inhibition. Methods Enzymol 252: 26–30. 3. Susek RE, Ausubel FM, Chory J (1993) Signal transduction mutants of Arabidopsis uncouple nuclear CAB and RBCS gene expression from chloroplast development. Cell 74: 787–799.
(6.69 MB TIF)

Figure S7 Confirmation of micro-array data by qRT-PCR. Expression of the chosen genes was monitored by qRT-PCR in Col-0 (light colours) or atips1-1 (dark colours) grown under SD (green bars) or under LD (blue bars). AtAct2 was used as internal control for signals normalization.
(0.21 MB TIF)

Figure S8 Down-regulation of GUN4 prevents lesion formation in the atips1-1 mutant. (A) Homozygous atips1-1 mutants (B) Homozygous atips1-1 mutants transformed with a construct encoding an articifial micro-RNA targeting GUN4. Scale bar  = 0.5 cm for both panels.
(5.25 MB TIF)

Table S1 List of metabolites analysed by GC-TOF-MS
(0.03 MB DOC)

Table S2 Differentially expressed genes in atips1-1 vs Col-0 under SD and LD conditions. Three analysis were performed: atips1-1 transcriptome was compared to that of Col-0 under short days (SD), and under long days (LD), and atips1-1 transcriptome under SD was compared to atips1-1 transcriptome under LD (SD/LD). For each set of experiment, the log2 ratio (Ratio) for differential expression and P values (Pval) are indicated (See methods for details). Below is the legend for color codes used for ratios and P values. Cells highlighted in green correspond to significantly down-regulated genes, cells highlighted in red correspond to significantly up-regulated genes. Cells highlighted in black correspond to genes that are not significantly differentially expressed (i.e. Pval >5%). AGI numbers highlighted in green correspond to probes that match two different genes, in that case, the AGI number of the other target is indicated inthe second column. AGI numbers highlighted in gray correspond to unannotated genes. Stars indicate genes that are represented by two different probes on the array.
(0.46 MB DOC)

Table S3 Differentially expressed genes in atips1-1 between SD and LD conditions. atips1-1 transcriptome under SD was compared to atips1-1 transcriptome under LD (SD/LD). Genes listed in this table were significantly up or down-regulated after transfer to LD, but were not differentially expressed in atips1-1 and Col-0. For each set of experiment, the log2 ratio (Ratio) for differential expression and P values (Pval) are indicated (See methods for details). Below is the legend for color codes used for ratios and P values. Cells highlighted in green correspond to significantly down-regulated genes, cells highlighted in red correspond to significantly up-regulated genes. Cells highlighted in black correspond to genes that are not significantly differentially expressed (i.e. Pval >5%). AGI numbers highlighted in green correspond to probes that match two different genes, in that case, the AGI number of the other target is indicated inthe second column. AGI numbers highlighted in gray correspond to unannotated genes.
(0.18 MB DOC)

Table S4 Genes putatively related to pathogen defence diferentially expressed in atips1-1. Two analysis are shown: atips1-1 transcriptome was compared to that of Col-0 under short days (SD), and under long days (LD). For each set of experiment, the log2 ratio (Ratio) for differential expression and P values (Pval) are indicated (See methods for details). Values highlighted in gray correspond to genes that are not significantly differentially expressed (i.e. Pval >5%). AGI numbers highlighted in green correspond to probes that match two different genes, in that case, the AGI number of the other target is indicated inthe second column. Stars indicate genes that are represented by two different probes on the array.
(0.03 MB DOC)

Table S5 Genes putatively related to oxidative stress diferentially expressed in atips1-1. Two analysis are shown: atips1-1 transcriptome was compared to that of Col-0 under short days (SD), and under long days (LD). For each set of experiment, the log2 ratio (Ratio) for differential expression and P values (Pval) are indicated (See methods for details). Values highlighted in gray correspond to genes that are not significantly differentially expressed (i.e. Pval >5%). Stars indicate genes that correspond to two different probes on the array.
(0.02 MB DOC)

Table S6 Differentially expressed chloroplastic genes in atips1-1 under SD and LD conditions. Three analysis were performed: atips1-1 transcriptome was compared to that of Col-0 under short days (SD), and under long days (LD), and atips1-1 transcriptome under SD was compared to atips1-1 transcriptome under LD (SD/LD). For each set of experiment, the log2 ratio (Ratio) for differential expression and P values (Pval) are indicated (See methods for details). Below is the legend for color codes used for ratios and P values. Cells highlighted in green correspond to significantly down-regulated genes, cells highlighted in red correspond to significantly up-regulated genes. Cells highlighted in black correspond to genes that are not significantly differentially expressed (i.e. Pval >5%). AGI numbers highlighted in green correspond to probes that match two different genes, in that case, the AGI number of the other target is indicated in the second column. AGI numbers highlighted in gray correspond to unannotated genes.
(0.03 MB DOC)
