# Supplementary figures and images for: Crosstalks between Myo-Inositol Metabolism, Programmed Cell Death and Basal Immunity in Arabidopsis
Source: PLoS One. 2009 Oct 8;4(10):e7364. doi: 10.1371/journal.pone.0007364 (PMC2754662; doi:10.1371/journal.pone.0007364)

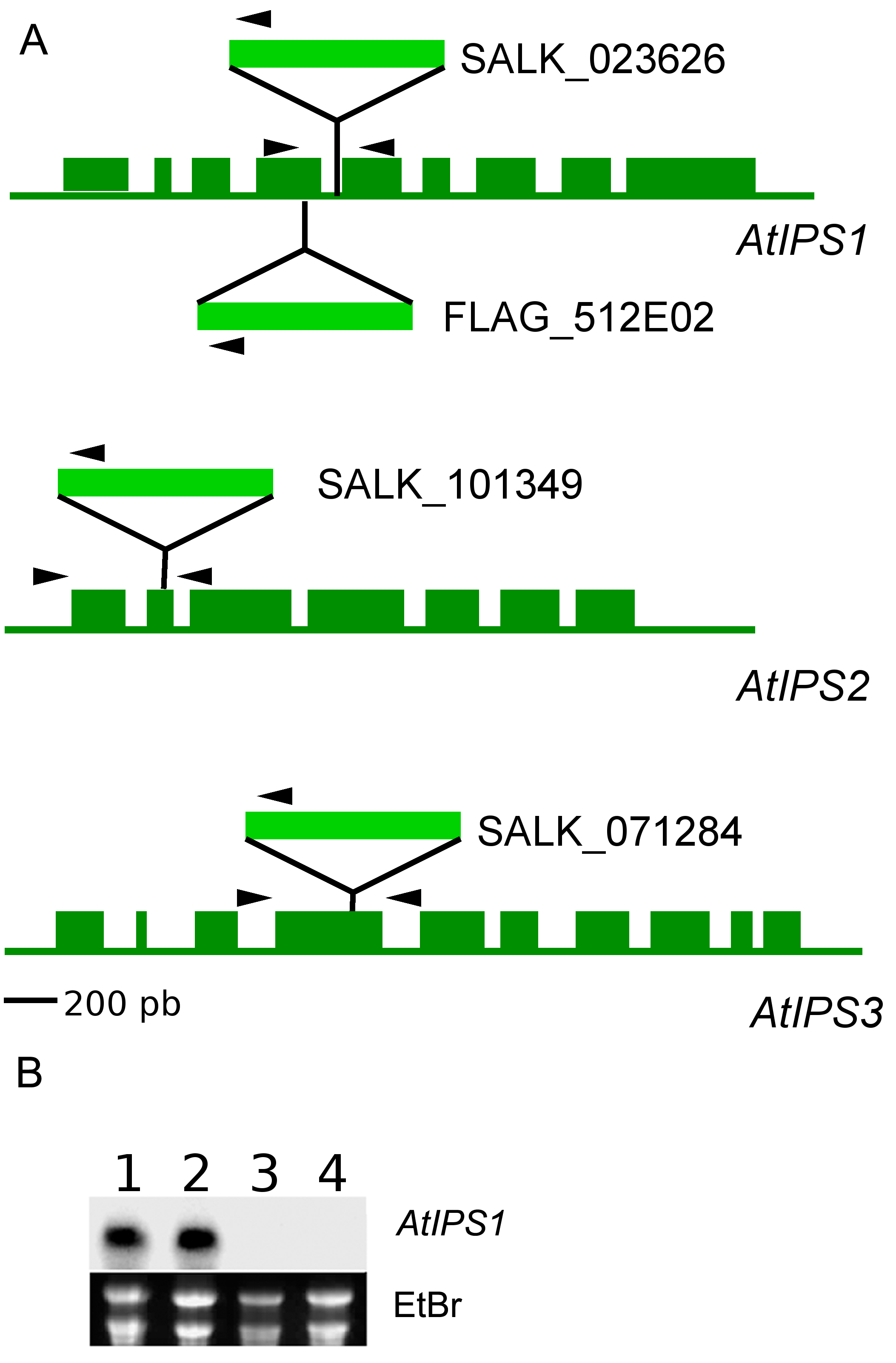

Supplement: Figure S1 — Structure of AtIPS genes and position of the T-DNA insertions. (A) Gene structure of AtIPS1, AtIPS2 and AtIPS3. Exons are represented as boxes and introns as lines. The position of T-DNA insertions in the mutants used in this study is indicated for each gene. Arrows represent the primers used for identification of homozygous mutants. (B) RNA gel blot analysis of total RNA isolated from wild-type (lanes 1, 2) atips1-1 (lane 3) and atips1-2 (lane 4) EtBr: ethidium bromide. (0.30 MB TIF) [file pone.0007364.s001.tif]

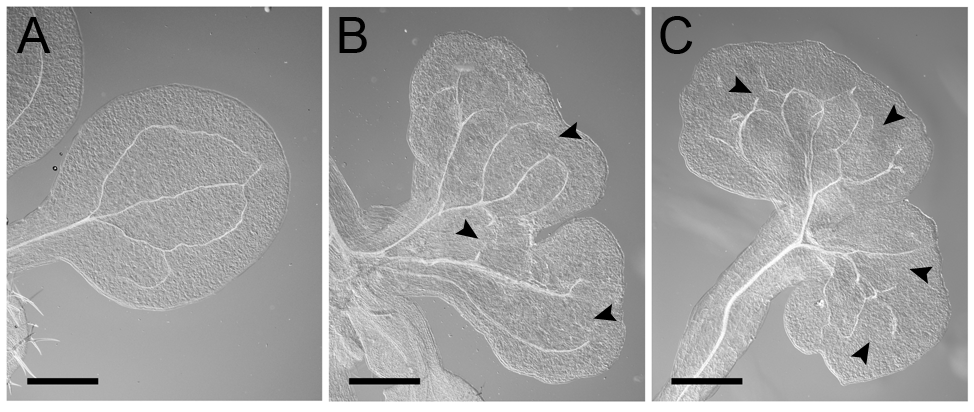

Supplement: Figure S2 — Cotyledons of atips1 mutants are deformed. Five day-old plantlets of the wild-type (A) and atips1-1 mutants (B, C) were fixated in ethanol/acetic acid (3∶1 v/v) and cleared by chloralhydrate treatment. Veins form a closed network in the wild-type, while this network is open in the mutant. Arrows indicate breaks in the vein network. (2.43 MB TIF) [file pone.0007364.s002.tif]

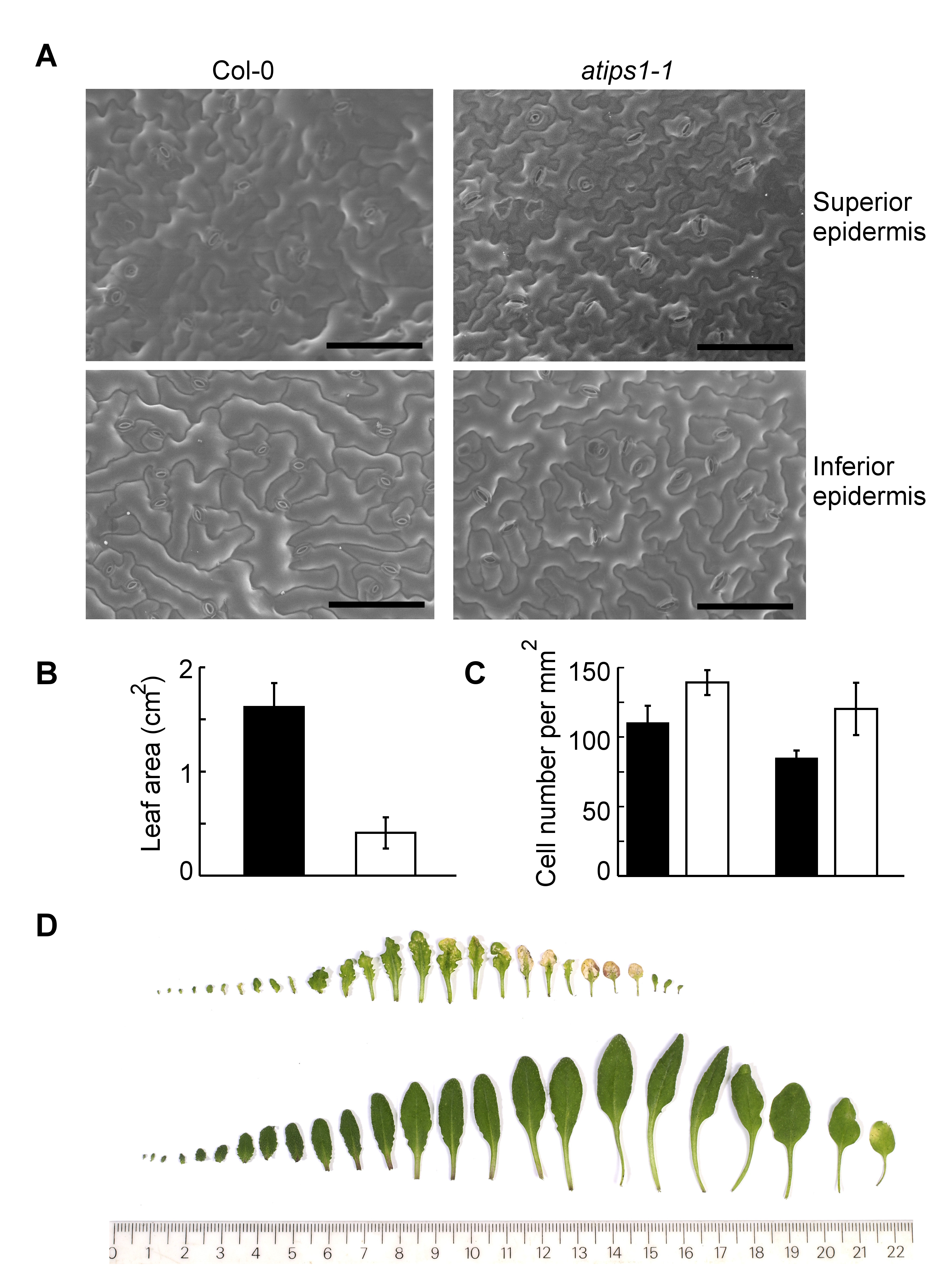

Supplement: Figure S3 — Disruption of AtIPS1 affects cell proliferation. (A) SEM image of wild-type and atips1 leaf epidermis. Scale bar = 100 µm. (B) Average leaf area in WT (black bars) and atips1 (white bars) plants. (C) Number of cells per surface unit in WT (black bars) and atips1 (white bars) plants for abaxial (left) and adaxial (right) epidermis. (D) Leaf size of representative atips1-1 and wild-type plants. (6.43 MB TIF) [file pone.0007364.s003.tif]

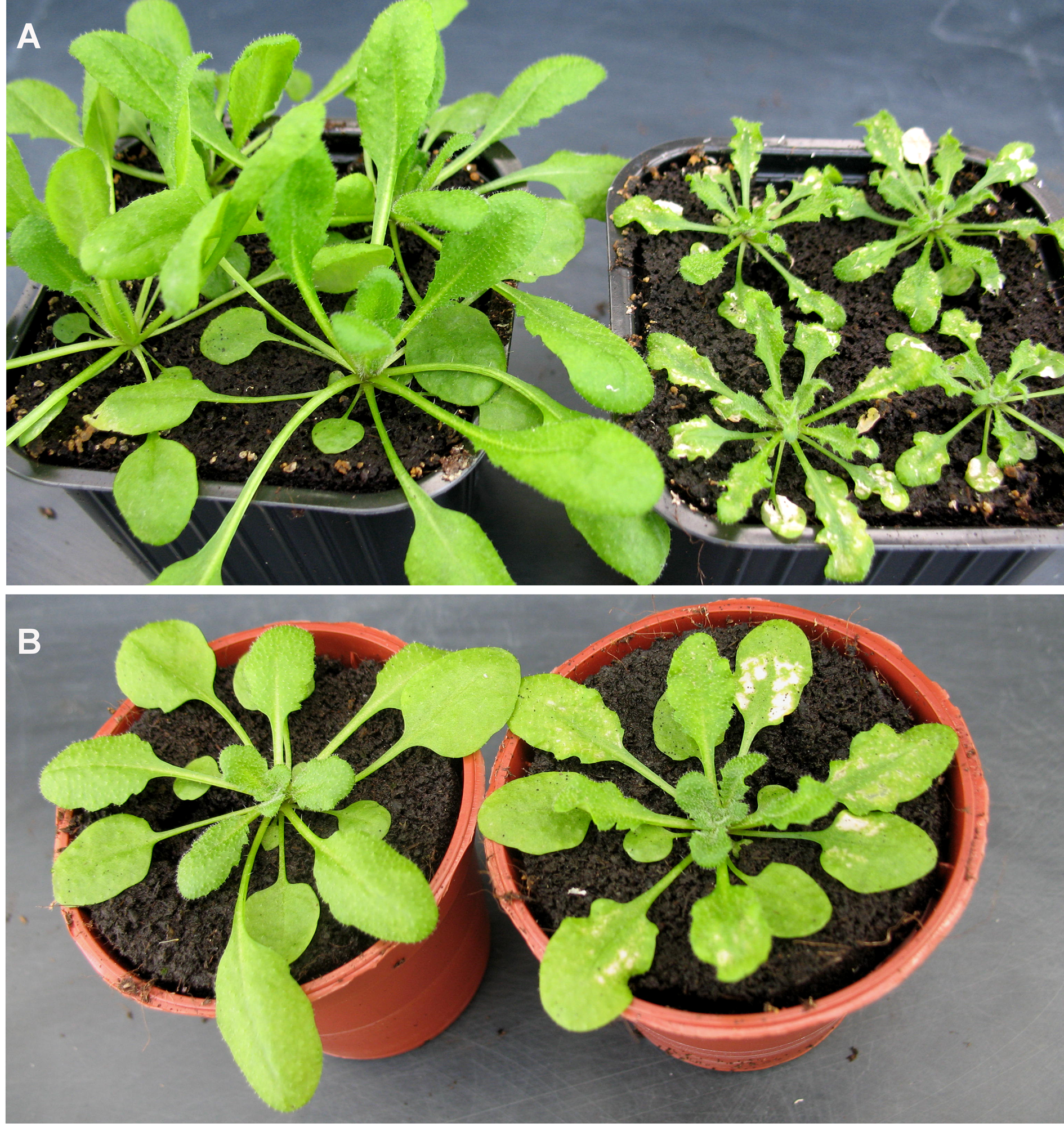

Supplement: Figure S4 — Phenotype of atips1-1 plants grown under SD and higher irradiance. Plants were grown under SD conditions. They were kept under low irradiance (45 µE/m2/s) for a month and transferred under LD at the same light intensity (A) or SD at higher irradiance (225 µE/m2/s) (B) for two weeks. Lesion formation occurred in both cases, but they spread more rapidly and plant growth was more affected in LD. (6.89 MB TIF) [file pone.0007364.s004.tif]

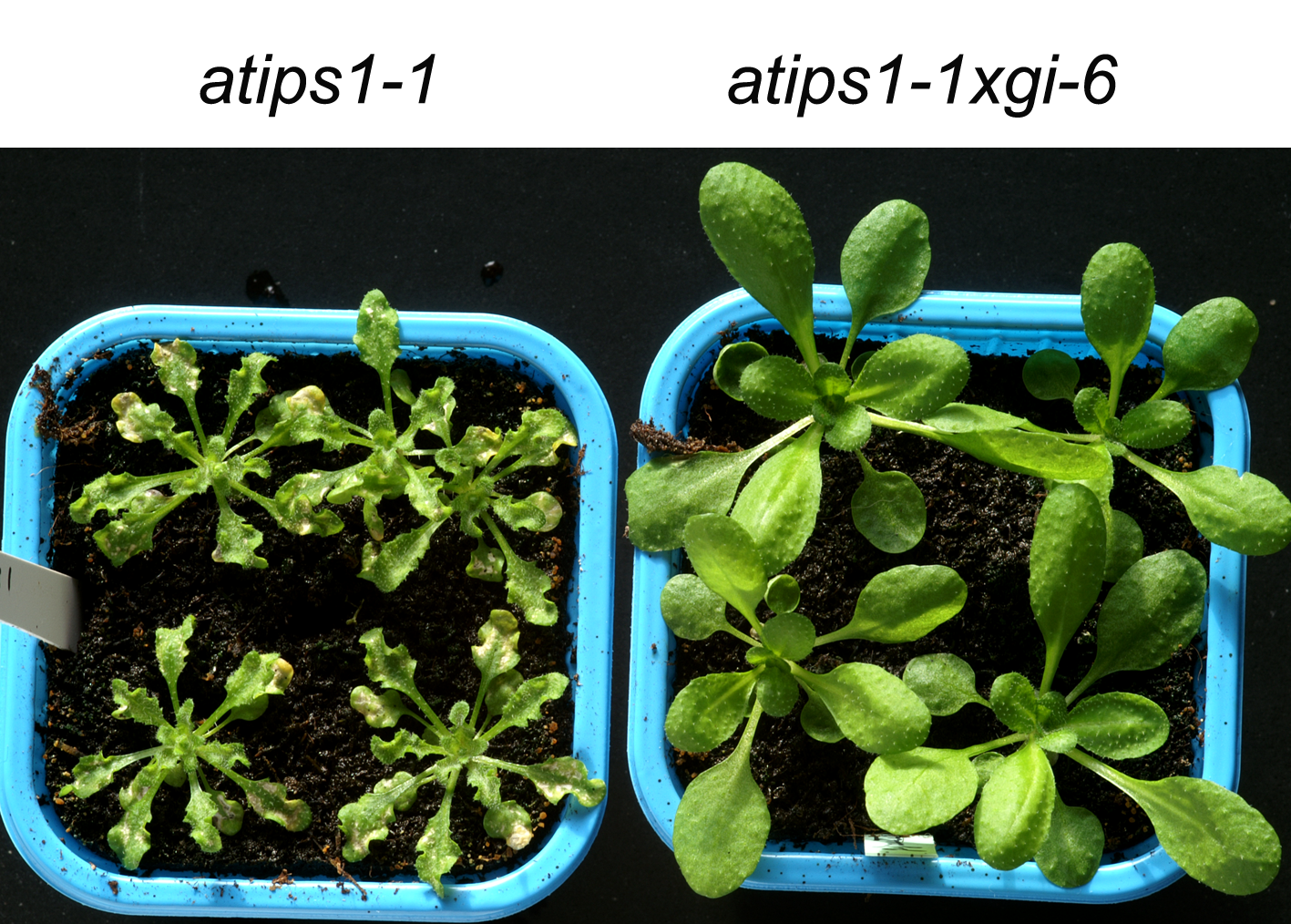

Supplement: Figure S5 — Lesion formation is drastically reduced in the atips1-1/gi-6 double mutant. Plants were grown under SD conditions. They were kept under low irradiance (45 µE/m2/s) for a month and transferred under LD at the same light intensity. The atips1-1 mutation induced lesion formation and growth inhibition in the Ler background. By contrast, atips1-1/gi-6 mutants form little or no lesions and grew normally, but showed delayed flowering like the gi-6 mutant (not shown). (4.33 MB TIF) [file pone.0007364.s005.tif]

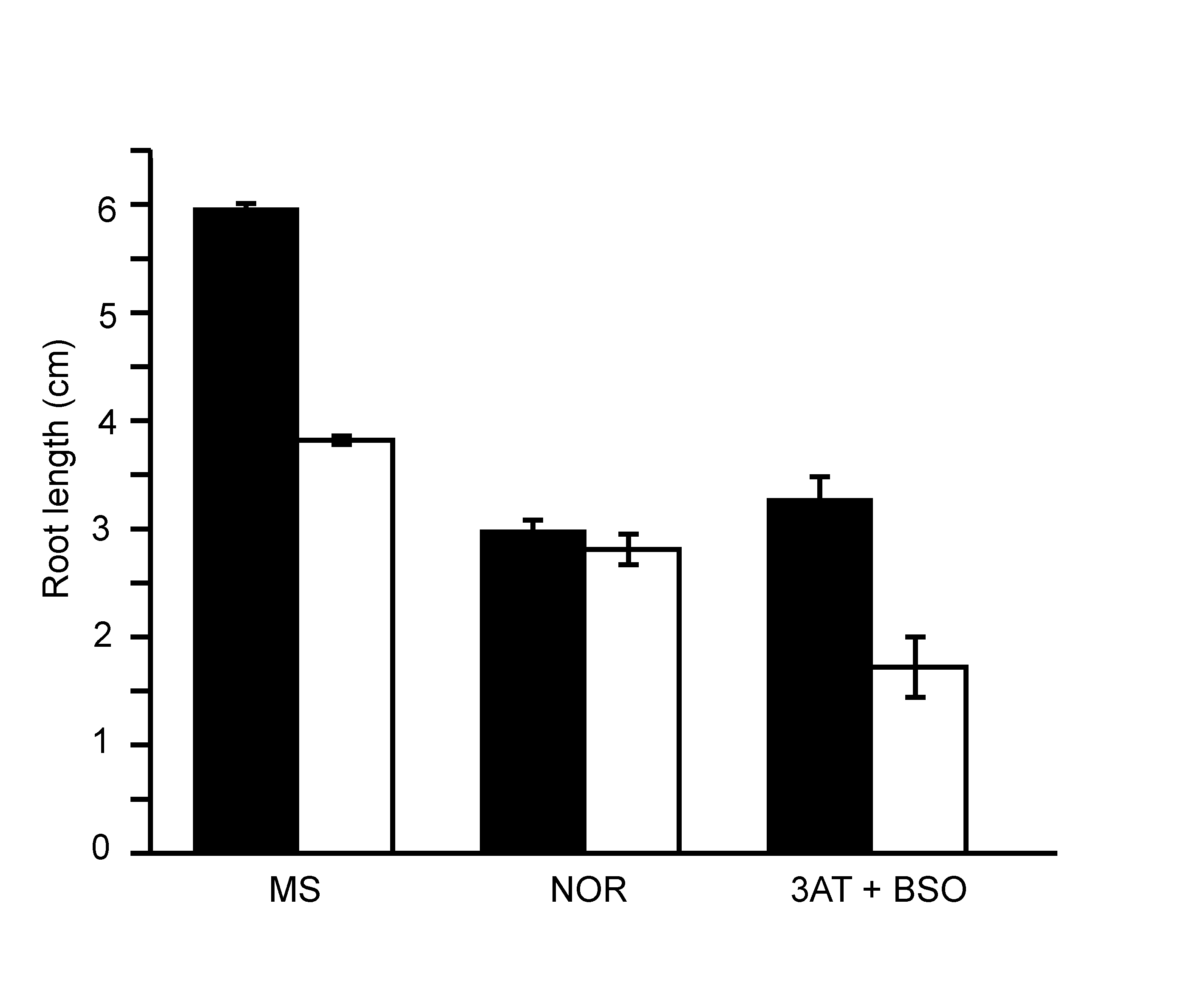

Supplement: Figure S6 — Oxidative stress tolerance is not reduced in atips1 mutants. Experiments were performed under SD conditions. Wild-type (black bars) and atips1-1 (white bars) plants were cultivated on 0.5× MS for 12 days and transferred to 0.5× MS medium (MS) or 0.5× MS medium containing norflurazon (NOR) or 3-amino-1, 2, 4-triazole (3AT) and DL-buthionine-(S,R)-sulfoximine (BSO). General oxidative stress was induced by treating plants with 3AT and BSO: 3AT is an inhibitor of catalase, and therefore generates H2O2 accumulation [1], while BSO inhibits gluthation biosynthesis, thus inhibiting this ROS scavenging pathway [2]. Norflurazon is an inhibitor of carotenoid biosynthesis: plants treated with norfluorazon suffer from photooxidation of the thylakoid membrane, treatment with norflurazon therefore generates oxidative stress preferentially in chloroplasts [3]. After one week, roots were measured. We observed a two-fold reduction in root-length for wild-type plants on both media and for atips1-1 on 3AT+BSO. By contrast, NOR treatment only resulted in a 1.3 fold reduction in root growth in the mutant, suggesting that atips1-1 may be more tolerant than the wild-type to norflurazon. 1. May MJ, Leaver CJ (1993) Oxidative Stimulation of Glutathione Synthesis in Arabidopsis thaliana Suspension Cultures. Plant Physiol 103: 621–627. 2. Meister A (1995) Glutathione biosynthesis and its inhibition. Methods Enzymol 252: 26–30. 3. Susek RE, Ausubel FM, Chory J (1993) Signal transduction mutants of Arabidopsis uncouple nuclear CAB and RBCS gene expression from chloroplast development. Cell 74: 787–799. (6.69 MB TIF) [file pone.0007364.s006.tif]

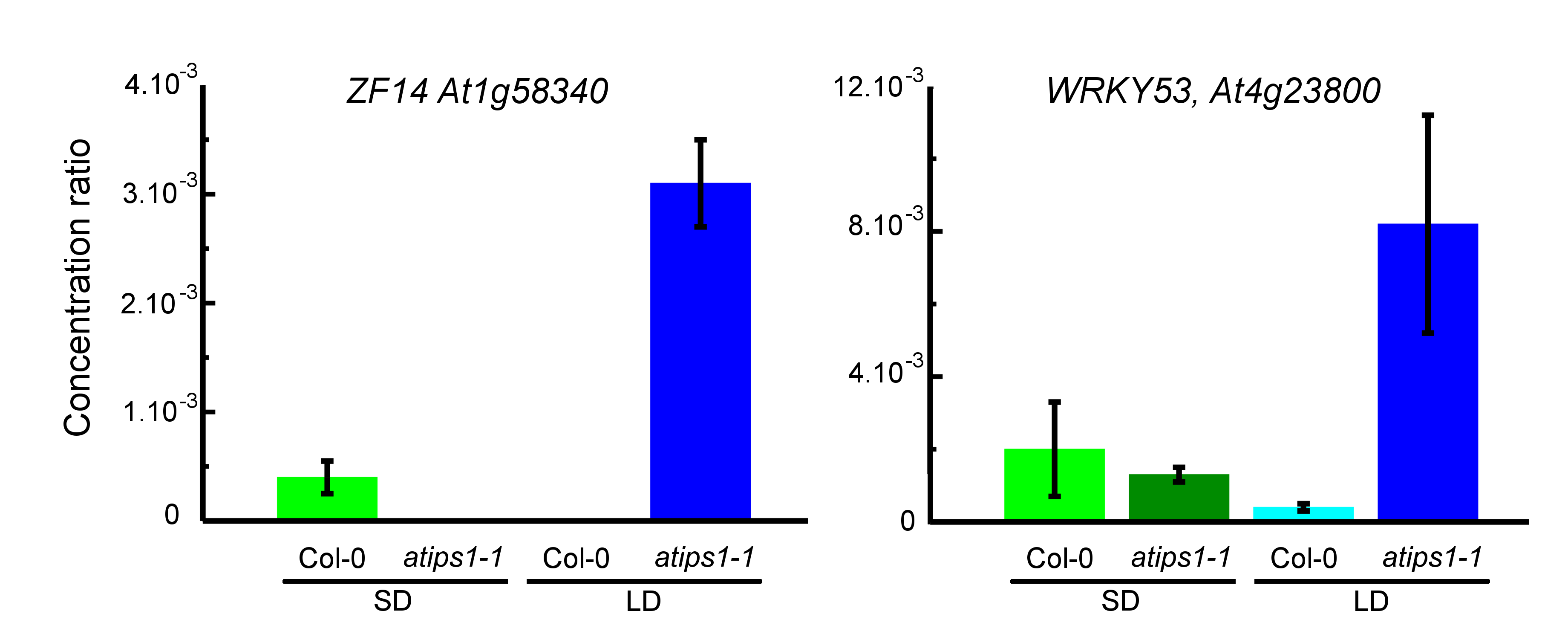

Supplement: Figure S7 — Confirmation of micro-array data by qRT-PCR. Expression of the chosen genes was monitored by qRT-PCR in Col-0 (light colours) or atips1-1 (dark colours) grown under SD (green bars) or under LD (blue bars). AtAct2 was used as internal control for signals normalization. (0.21 MB TIF) [file pone.0007364.s007.tif]

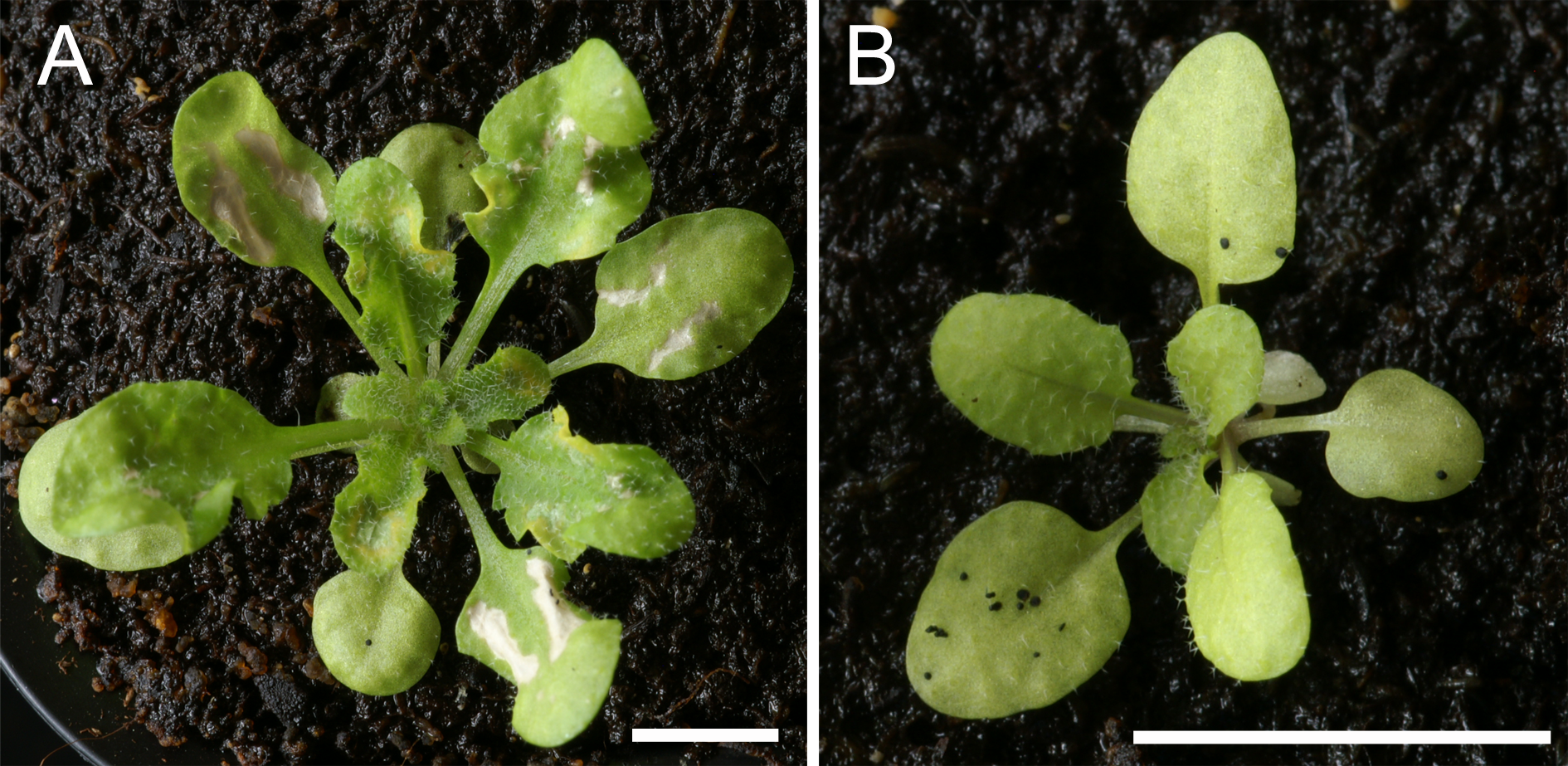

Supplement: Figure S8 — Down-regulation of GUN4 prevents lesion formation in the atips1-1 mutant. (A) Homozygous atips1-1 mutants (B) Homozygous atips1-1 mutants transformed with a construct encoding an articifial micro-RNA targeting GUN4. Scale bar = 0.5 cm for both panels. (5.25 MB TIF) [file pone.0007364.s008.tif]
