# Supplementary material for: Crosstalks between Myo-Inositol Metabolism, Programmed Cell Death and Basal Immunity in Arabidopsis
Source: PLoS One. 2009 Oct 8;4(10):e7364. doi: 10.1371/journal.pone.0007364 (PMC2754662; doi:10.1371/journal.pone.0007364)
Supplement: Table S1 — List of metabolites analysed by GC-TOF-MS (0.03 MB DOC) [file pone.0007364.s009.doc]

| **Amino-acids** | **Sugars and polyols** | **Usual organic acids** | **Lipids** | **Others** |
| --- | --- | --- | --- | --- |
| α-alanine | di-galactosilglycerol | Ascorbic acid | β-sitosterol | Pentonic acid |
| Asparagine | Fructose | Citramalic acid | Linoleic acid | Pipecolic acid |
| Aspartic acid | Galactinol | Citric acid | Linolenic acid | Putrescine |
| β-alanine | Galactose | Fumaric acid | Oleic acid | Spermidine |
| Cysteine | Galactosylglycerol | Glutaric acid | Palmitic acid | Shikimic acid |
| GABA | Glucose | Glyceric acid | Squalene | T-Sinapinic acid |
| Glutamine | Ribose | Glycolic acid | Stearic acid | Urea |
| Glutamic acid | Sucrose | Maleic acid |  |  |
| Glycine | Xylose | Malic acid |  |  |
| Homo-Serine |  | Pyruvic acid |  |  |
| Isoleucine |  | Succinic acid |  |  |
| Leucine |  |  |  |  |
| Lysine |  |  |  |  |
| Methionine |  |  |  |  |
| O-Acetyl-Serine |  |  |  |  |
| Ornithine |  |  |  |  |
| Phenyl-alanine |  |  |  |  |
| Proline |  |  |  |  |
| Pyro-glutamate |  |  |  |  |
| Serine |  |  |  |  |
| Threonine |  |  |  |  |
| Tryptophane |  |  |  |  |
| Tyrosine |  |  |  |  |
| Valine |  |  |  |  |
